# Small-molecule modulation of the p75 neurotrophin receptor inhibits a wide range of tau molecular pathologies and their sequelae in P301S tauopathy mice

**DOI:** 10.1186/s40478-020-01034-0

**Published:** 2020-09-05

**Authors:** Tao Yang, Harry Liu, Kevin C. Tran, Albert Leng, Stephen M. Massa, Frank M. Longo

**Affiliations:** 1 Department of Neurology and Neurological Sciences, Stanford University School of Medicine, 300 Pasteur Drive, Room H3160, Stanford, CA 94305 USA; 2 Department of Neurology, San Francisco Veterans Affairs Health Care System and University of California, San Francisco, 4150 Clement St., San Francisco, CA 94121 USA

## Abstract

In tauopathies, phosphorylation, acetylation, cleavage and other modifications of tau drive intracellular generation of diverse forms of toxic tau aggregates and associated seeding activity, which have been implicated in subsequent synaptic failure and neurodegeneration. Suppression of this wide range of pathogenic species, seeding and toxicity mechanisms, while preserving the physiological roles of tau, presents a key therapeutic goal. Identification and targeting of signaling networks that influence a broad spectrum of tau pathogenic mechanisms might prevent or reverse synaptic degeneration and modify disease outcomes. The p75 neurotrophin receptor (p75^NTR^) modulates such networks, including activation of multiple tau kinases, calpain and rhoA-cofilin activity. The orally bioavailable small-molecule p75^NTR^ modulator, LM11A-31, was administered to tau^P301S^ mice for 3 months starting at 6 months of age, when tau pathology was well established. LM11A-31 was found to reduce: excess activation of hippocampal cdk5 and JNK kinases and calpain; excess cofilin phosphorylation, tau phosphorylation, acetylation and cleavage; accumulation of multiple forms of insoluble tau aggregates and filaments; and, microglial activation. Hippocampal extracts from treated mice had substantially reduced tau seeding activity. LM11A-31 treatment also led to a reversal of pyramidal neuron dendritic spine loss, decreased loss of dendritic complexity and improvement in performance of hippocampal behaviors. These studies identify a therapeutically tractable upstream signaling module regulating a wide spectrum of basic mechanisms underlying tauopathies.

**Electronic supplementary material:**

The online version of this article (10.1186/s40478-020-01034-0) contains supplementary material, which is available to authorized users.

## Introduction

In tauopathies, pathological tau phosphorylation and other post-translational modifications (PTMs), along with tau misfolding, lead to formation of toxic intermediates involving a sequence of tau monomers, oligomers, protofibrils and filamentous species, resulting in the accumulation of neurofibrillary tangles (NFTs). NFTs are considered to be inert, while pre-NFT intermediates promote tau seeding [33], loss of dendritic spines and synapses [24, 29, 31] and microglial activation [42, 54]. Filamentous forms of tau identified by PET tracers demonstrate a notable longitudinal association with grey matter degeneration [40]. Numerous challenges in tauopathy therapeutics have been identified, including: target selection and prioritization among multiple mechanisms, including tau misfolding, aggregation and spreading; the molecular diversity of pathological tau isoforms [16]; access to tau intracellularly, where toxic species are formed and where critical degenerative events occur; and finally, avoiding chronic interference in physiological roles of tau [34, 66]. Further, when pathology is initiated by tau mutations, therapies must overcome mutation-induced protein misfolding and aggregation. Thus, an important goal is the identification of ‘upstream’ or ‘proximal’ targets through which engagement would sufficiently inhibit multiple pathological tau molecular processes to reduce tau pathology, and moreover prevent misfolding and aggregation of mutant human tau.

Therapeutic strategies demonstrating efficacy in tauopathy mouse models include antibody targeting of specific tau species, antisense-mediated inhibition of tau expression, and small molecule-mediated inhibition of specific tau kinases, acetyltransferases, proteases or aggregation [59]. Several of these have entered human tauopathy trials and while largely at early stages, none have demonstrated efficacy as yet [5, 9, 14].

A target with the potential to affect a broad array of tauopathy-relevant neurodegenerative mechanisms is the p75 neurotrophin receptor (p75^NTR^). p75^NTR^ modulates multiple tau-related signaling pathways, including those regulating tau kinases [10, 16, 22, 60, 61], caspase activity promoting tau cleavage [10, 39] and GTPase signaling regulating dendritic spine stability [35, 43] and tau phosphorylation [76]. In its unliganded ‘default’ state, or in the presence of proneurotrophin ligands, p75^NTR^ stimulates degenerative signaling, including excess activation of jun kinase (JNK) and RhoA GTPase. In other settings, p75^NTR^ promotes survival signaling [60]. LM11A-31 is a small-molecule, orally bioavailable p75^NTR^ ligand which functions as a receptor modulator to downregulate degenerative signaling and upregulate survival signaling [47]. In studies employing the APP-Lon/Swe amyloid-based mouse model, oral administration of LM11A-31 reduced phosphorylation and misfolding of non-mutant, mouse tau [55] and degeneration of dendrites and dendritic spines [38, 55, 67]. LM11A-31 is currently undergoing assessment in a phase 2a exploratory endpoint AD clinical trial (NCT03069014).

In mouse models expressing mutant human tau, accumulation of misfolded tau triggers excess activation of tau kinases, including JNK, cdk5 and GSK3β, resulting in hyperphosphorylation of tau [2, 64, 80]. Crossing p75^NTR^ exon III knockout mice with pR5 P301L tauopathy mice led to reduced activation of tau kinases and decreased tau phosphorylation and caspase activity [45]. Given the p75^NTR^-modulating properties of LM11A-31, we conjectured that it might significantly lessen tauopathic disease pathology and improve function. In the present study, we find that LM11A-31 diminishes upstream signaling relevant to tau modification, including calpain and tau kinase activities, and mitigates disease correlates and outcomes in these mice including accumulation of human pathological tau species, tau seeding activity, synaptic degeneration, microglial activation, behavioral impairment and early death in PS19 (P301S) tauopathy mice. These results serve to identify a therapeutically tractable ‘upstream’ signaling module regulating a broad spectrum of tau molecular pathology.

## Materials and methods

### Animals, drug administration and testing schedule

PS19 mice express a 1N4R human tau cDNA insert with a TauP301S mutation resulting in levels of human tau 5-fold greater than endogenous mouse tau [79]. Male transgenic (Tg) mice along with age- and strain-matched non-transgenic (Ntg) mice were purchased from The Jackson Laboratory (008169, B6;C3-Tg(*Prnp*-MAPT*P301S)PS19Vle/J). Male mice were utilized as they develop more abundant tau pathology than females [78, 83]. Mice had access to food and water ad libitum and were maintained on a 12 h light/dark cycle. Animals were separated by genotype and housed in separate cages within common cage racks. Animal procedures were conducted in accordance with the National Institutes of Health Guide for the Care and Use of Laboratory Animals using protocols approved by the Institutional Animal Care and Use Committee at Stanford University. LM11A-31 in a sulphate salt form [(2S,3S)-2-amino-3-methyl-N-(2-morpholinoethyl) pentanamide] was custom manufactured by Olon Ricerca Biosciences (Concord, OH) at > 99% purity. LM11A-31 (C31) dissolved in sterile water (5 mg/ml) or vehicle (V, sterile water alone), was administered via oral gavage at a dose of 50 mg/kg (equivalent to 30 mg/kg of the free base form) once daily, five days per week (Monday-Friday), for 3 months beginning at 6 months of age. At age 9 months, one hour following the final dose, mice were perfused with PBS followed by brain tissue harvesting. Each brain was divided into halves, one for morphological and one for biochemical studies.

### Protein extraction and Western blot analysis

For total soluble protein extraction, frozen hippocampal tissue was homogenized in RIPA lysis buffer (150 mM NaCl; 1% NP-40; 50 mM Tris, pH7.4; 1 mM EDTA; 10% glycerol, 1 mM PMSF) with one tablet of complete protease and phosphatase inhibitors (Thermo Fisher Scientific, Waltham, MA, Cat# A32959) [48]. Homogenates were spun at 10,000 g for 10 min and supernatants collected.

Sarkosyl-insoluble tau protein extraction was carried out as previously described [63]. Frozen hippocampal tissue was homogenized in cold extraction buffer [10 mM Tris–HCl, pH 7.4, 800 mM NaCl, 1 mM EGTA, 0.1 M phenylmethylsulphonyl fluoride, 10% sucrose and one tablet of complete protease and phosphatase inhibitors cocktail (Thermo Fisher Scientific). Homogenates were spun at 6000 g for 20 min and supernatants were collected and then incubated with 1% sarkosyl for 1 h at room temperature. After 1 h centrifugation at 80 000 g, pellets were collected and resuspended in 50 mM Tris–HCl, pH 7.4.

Synaptosome-enriched fraction extraction was performed using an established method [72] with modifications. Ice-cold sucrose buffer (TEVP buffer: 10 mM Tris–HCl, pH 7.4, 320 mM sucrose, 4 mM HEPES, 1 mM Na3VO4, 1 mM EDTA) was rapidly added to mouse hippocampal tissue followed by sonication for 10 s and incubation on ice for 30 min. Whole homogenates were centrifuged to pellet the nuclear/cytoskeletal fraction (4 °C, 1000 g, 12 min, P1) and the resulting supernatants (S1) were centrifuged to pellet the synaptosomal fraction (4 °C, 25 000 × *g*, 30 min, LP1). Synaptosome samples were resuspended in a detergent-based protein lysis buffer (100 mM NaCl, 20 mM HEPES, 1 mM EDTA, 1 mM dithiothreitol, 1.0% Triton, 1 mM Na3VO4, one tablet of complete protease and phosphatase inhibitors cocktail (Thermo Fisher Scientific).

Protein concentration was measured using the Precision Red Advanced Protein Assay (Cytoskeleton, Inc., Denver, CO). Protein extractions were stored at − 80 °C. For Western blotting, 20–40 µg aliquots of protein extract from each sample were run in each lane on precast NuPAGE 4–12% Bis–Tris Gels for SDS-PAGE (Thermo Fisher Scientific) then transferred to PVDF membranes. After blocking with 5% nonfat dried milk at room temperature for 1 h, membranes were probed overnight at 4 °C with one of the following antibodies: AT180 and AT270 (1:1000, Thermo Fisher Scientific); HT7 (1:5000, Thermo Fisher Scientific); Tau^Acetyl K280^ (1:1000, AnaSpec, Fremont, CA). PHF-1 and MC1 (each 1:1000, gifts from Peter Davies); p-cofilin^Ser3^, cofilin, p-JNK^Thr183/Tyr185^, JNK, p-GSK3β^Ser9^, GSK3β, pERK^Thr202/Tyr204^, ERK and Tau (D1M9X) XP^®^ Rabbit mAb (1:1000, Cell Signaling, Danvers, MA); α-fodrin and p35/p25 (1:1000; Santa Cruz Biotechnology, Santa Cruz, CA); actin (1:10,000; Sigma, St. Louis, MO). Secondary antibodies were either horseradish peroxidase (HRP)-conjugated anti-rabbit IgG (1:10,000; Thermo Fisher Scientific) or anti-mouse IgG (1:10,000; Agilent DAKO, Santa Clara, CA) ECL (GE Healthcare, Sunnyvale, CA). Band densities were measured using Un-Scan-It gel software (Ver. 6.14, Silk Scientific. Inc, Orem, UT).

### Thioflavin S and Immunostaining

Brains were post-fixed in 4% paraformaldehyde and sliced at 40 µm on a freezing microtome. For Thioflavin S analysis, sections were mounted onto frosted slides and dried overnight at room temperature. Before staining, sections were permeabilized in 80% ice-cold methanol and pre-treated in 0.4% TritonX-100 for 20 min. Slides were then dipped in distilled water for 10 min, followed by one dip in 1% Thioflavin S (filtered) for 8 min, ten dips in 80% ethanol for 3 s each, another dip in 1% Thioflavin S for 8 min, and ten dips in 95% ethanol for 3 s. Slides were then washed in double distilled water three times, 5 min each in the dark. Sections were coverslipped with Prolog Gold Antifade Reagent with DAPI (Thermo Fisher Scientific). For immunostaining, sections were incubated with the primary antibodies, AT8 (anti-mouse; 1:500, Thermo Fisher Scientific), Iba1(anti-rabbit; 1:1000, Wako, Richmond, VA) or CD68 (anti-rat; 1:750, Bio-Rad, Hercules, CA) in 3% donkey serum/bovine serum albumin in 1X TBS 0.4% TritonX-100 overnight at 4 °C, followed by a three-hour incubation at room temperature with fluorescent-conjugated secondary antibodies (1:400 anti-mouse FTIC 488, 1:400 anti-rabbit CY3 550, Thermo Fisher Scientific). Sections were mounted and cover slipped using Prolog Gold Antifade Reagent with DAPI.

### Thioflavin S and Immunostaining Quantification

Pairs of 40 µm brain sections, each 640 µm apart, from each mouse were assessed. 40x magnification z-stack images (step size 0.7 µm) of mounted hippocampal sections, including the pyramidal and granule cell layers, were obtained using a Leica DM550 confocal microscope (Leica Microsystems Inc., Buffalo Grove, IL). Using sections with positive signals, the laser power, gain and offset settings were adjusted using LAS X viewer software (Leica) to optimize the range of signal capture. Identical settings were then applied to all subsequent image files for a given staining set, each including all four genotype/treatment conditions. Image files were converted to 8-bit greyscale and maximum intensity projections were generated using FIJI/ImageJ. Using the rolling ball algorithm, background was automatically subtracted, and contours of the pyramidal and granule cell layers of CA1, CA3, and dentate gyrus of the left hippocampus were drawn with the freehand selection tool. A threshold range was visually selected for each staining set to optimize the range of signal capture. The same threshold range was applied to all image files in the given staining set before performing the “analyze particles” task to determine the percent area of signal within the contour. The average percent area coverage of two sections was used as the final value for AT8, Iba1, CD68 or Thioflavin S signal for each mouse.

### Hippocampal volume analysis

Starting at the rostral boundary of the dorsal hippocampus (bregma − 1.06 mm), 12–15 40 µm sections were obtained at 160 µm intervals until reaching the caudal boundary (bregma − 2.54 mm). Nissl-stained sections were imaged at 5 × magnification using a Zeiss AXIO Imager M.2 microscope. The hippocampus in each section was highlighted using the Cavalieri Estimator in Stereo Investigator (MBF Bioscience, Williston, VT) and estimated volume for each series of sections was obtained in µm^3^ and converted by the software protocol to mm^3^.

### Tau Seeding Assay

Hippocampal extracts were prepared as described by Furman et al. [20]. Tau seeding activity of tissue extract was determined using HEK293T cells stably transfected to express the repeat domain of P301S tau fused with either CFP or YFP [20, 30]. 12-well plates containing glass cover slips were treated with poly-l-lysine solution (Sigma) for 1 h at 37 °C and washed three times with PBS. Cells were plated at a density of 420,000–450,000 cells in 1 mL culture media (DMEM with 10% FBS) per well and incubated at 37 °C. After reaching 60–70% confluency in 18–20 h, cells were transfected with protein lysate from mouse hippocampal homogenates. Three independent transfection experiments were conducted; each consisting of 4 mouse treatment groups (Ntg-V, Tg-V, Ntg-C31, Tg-C31) with 4 mice per group. Individual mouse lysates for each study were assessed in two independent assays. Transfection complexes were formed by adding 40 µg protein lysate in TBS buffer to 13 µL of Lipofectamine 2000 (Invitrogen) with opti-MEM (Gibco) to a total volume of 50 µL per transfection condition. Transfection mixtures were allowed to sit undisturbed for 20 min before being added drop-wise to each well. For the controls testing acute effects of LM11A-31, a final concentration of 100 nM LM11A-31 was added to wells with Ntg-V and Tg-V hippocampal lysates during transfection. Transfection complexes remained in wells for 7–8 h and were then replaced with warm media. Cells were allowed to recover for ~ 16 h and then fixed with 4% paraformaldehyde for 15 min. Glass cover slips containing cells were mounted with Prolong Gold Antifade reagent with DAPI and set overnight. Three z-stacks per well with a step size of 1 µm and 12–24 slices each were imaged on the Leica confocal microscope using the 40x oil immersion lens with 405 (DAPI) and 488 (FRET) nm laser lines. For quantification, 3D objects in the entire z-stack corresponding to DAPI or FRET channels were created using the Imaris x64 9.3.1 (Bitplane Inc. Concord, MA) surface function, with a 30- and 42-unit (arbitrary voxel intensity units) threshold minimum respectively. Object count and total volume were obtained for DAPI and FRET. Percent FRET-positive cells was determined by running one representative slice containing the highest number of nuclei from each z-stack through the positive cell detection function in the QuPath 0.2.0 software with thresholds set at 8 units for detection of nuclei and 90 units for FRET positive cells. Total cell number was determined based on the number of nuclei. Cell boundaries were determined using a cell expansion parameter of 5 µm from the nucleus. Measurements were averaged across three z-stacks per well with two independent wells per mouse tissue lysate sample and four mice per group.

### Survival analysis

Survival analysis was performed using two independent approaches. In the first, mouse survival was recorded as part of the age 6–9-month treatment cohorts. Mice surviving through the end of the 3-month treatment period at 9 months were sacrificed and censored in the analysis at that point. In a separate study, treatment with vehicle or LM11A-31 was initiated at age 6 months and continued until mice required life-prolonging therapy, were moribund or dead, all considered equivalent primary endpoints. Animals were monitored seven days per week throughout each study.

### Modified Golgi staining

Mice were perfused with PBS and their brains were rapidly removed and immersed in modified Golgi-Cox staining solution (developed and provided by Deqiang Jing and Francis Lee, Cornell University) for 9 days at room temperature in the dark. They were then incubated in 30% sucrose in dH2O at 4 °C for 72 h with the solution changed after the first 12 h. Brains were cut into 150 μm sections using a vibratome and mounted onto slides coated with 0.3% gelatin. After drying briefly, slides were dipped in 40% sucrose three times and allowed to air dry for 72 h in the dark. After dH2O washes, sections were stained with developing solution, washed again, dehydrated through graded ethanols, immersed in xylene, and then coverslipped using DPX mounting medium.

### Analysis of dendrites and spines

Golgi-labeled pyramidal neurons, dendrites and spines were traced by an experimenter blinded to genotype/treatment, and reconstructed and enumerated using Neurolucida and NeuroExplorer software (MBF Bioscience). Following Neurolucida protocol guidelines, neurons in the CA1 region of the hippocampus displaying intense staining of dendritic arborizations and allowing unambiguous identification of dendritic spines were chosen for reconstructions. Spines were defined as protrusions perpendicular to the dendritic shaft measuring 0.5–2 μm in length and possessing a clear spine neck or head.

### Behavioral tests

Behavior testing and interpretation of results were performed in the Stanford Behavioral and Functional Neuroscience Laboratory. Animals were separated by genotype and housed in groups at a standard temperature (22 ± 1 °C) in a reverse-cycle, light-controlled environment (lights on from 8:30 PM to 8:30 AM). Groups were pseudo-randomized using baseline activity chamber performance and body weight [4]. Animals were habituated to the testing area for at least 1 h before testing and all testing was performed during the daily dark cycle. Apparatuses were cleaned with 1% Vikron solution between subjects unless otherwise specified.

#### Activity chamber

Activity Chamber testing was used to assess general activity levels, gross locomotor activity and exploration behavior. Testing took place in an Open Field Activity Arena (Med Associates Inc., St. Albans, VT., Model ENV-515) equipped with three planes of infrared detectors (Ethovision, Noldus Information Technology, Wageningen, Netherlands), inside a sound attenuating chamber (Med Associates Inc., MED-017M-027). The arena was 43 cm (L) × 43 cm (W) × 30 cm (H) and the sound attenuating chamber was 74 cm (L) × 60 cm (W) × 60 cm (H). Each mouse was placed in a corner of the testing arena and allowed to explore for 10 min. Parameters measured included distance moved, ambulatory time, vertical count, vertical time, velocity and time spent in the periphery and center of the arena. The periphery was defined as the zone ≥ 5 cm away from the wall of the arena. The test was performed in the dark inside sound attenuated chambers. For each mouse, a baseline trial was conducted at age 6 months prior to dosing and trials were repeated at 2, 6, and 11 weeks during the dosing 3-month period.

#### Elevated Plus-Maze

The Elevated Plus-Maze was used to examine anxiety-like behavior. The maze was made of acrylonitrile butadiene styrene (ABS) plastic and had two open arms and two closed arms, each 30 cm long and 5 cm wide. The center area where the open and closed arms intersect was 5 cm × 5 cm. The open arms had 2 mm lips at the edges and the closed arms had 15 cm opaque walls. The maze was elevated 50 cm and surrounded by privacy blinds during testing. The maze was illuminated to 7 Lux using red light throughout the assessment period. Each mouse was released in the center of the maze for a 5-min exploration period recorded using Ethovision XT (Noldus Information Technology, Wageningen, the Netherlands) tracking software. Test parameters included duration and frequency in each zone of the maze. The experiment was conducted during the 6th week of the 3-month dosing period.

#### Novel place recognition (nPR) and novel object recognition (NOR)

NPR/NOR testing was conducted in an open-top plastic arena 52 cm (L) × 52 cm (W) × 40 cm (H). A white index card 12.7 cm (L) × 7.62 cm (W) was positioned on one wall as visual cue. Testing was conducted over 3 days. On the first day (habituation), each mouse was placed at the center of the empty arena, allowing free exploration for 10 min. NPR training and testing was conducted on the second day. For NPR training, each mouse was placed at the center of the arena, which contained three identical objects located 10 cm from the corner of arena wall. At the end of the 10-minute training session, each mouse was returned to the home cage for 3–4 min, followed by NPR testing. For NPR testing, one of the objects was moved to the previously empty corner. Mice were placed into the center of the arena, observed for 5 min and returned to home cage. NOR assessment was conducted on the third day. For NOR testing, one of the three objects was removed and replaced with a different object and mice were again placed into the center of the arena for a 5-minute trial. The object replaced was different from the object used in the NPR testing. The placement of objects in NPR and the object replaced in NOR were pseudorandomized across different subjects. The trials were recorded with an automated Ethovision XT tracking system. Interaction was defined as nose location within 2 cm from the object. The interaction time during NPR training was tracked by the Ethovision system while the interaction times during NPR and NOR testing were hand scored by the experimenter who was blinded to the treatment group and genotype. Discrimination index was calculated as the time exploring the novel object minus the time exploring the familiar object, divided by the total time of exploration. The experiment was conducted during the 7 ×  week of the 3-month dosing period. By protocol, mice with low interaction with objects (< 1 s total interaction time for all objects combined during the testing session) were excluded from data analysis. A total of two Ntg-V mice were excluded due to low interaction with objects during NPR testing.

#### Morris water maze (MWM)

Spatial learning and memory testing were conducted using the water maze task [4]. For Hidden Platform Training (HPT), each mouse underwent a series of 4 trials, approximately 30–60 min apart in a large dark-colored tank (172 cm in diameter) filled with opaque water at a temperature of 22.0 ± 1.5 °C. Nontoxic tempera paint was used to render the water opaque. A 17 cm diameter circular platform was submerged 0.75–1 cm below the water surface and placed in one of the four quadrants of the pool (Quadrant 2). Release points were pseudorandomized to avoid directional bias. For each trial, each mouse was allowed a maximum of 60 s to find the submerged platform. The experimenter guided the mouse to the platform if they failed to find the platform within 60 s. After remaining on the platform for 10 s, the mouse was removed from the platform and placed in a dry cage with clean paper towel. For training sessions, this process was repeated 4 times for each animal per day for 4 consecutive days. Two cohorts of mice underwent training and one cohort was excluded due to inability to learn the task during the training sessions. For the remaining cohort, on the following day after the last training trial (~ 24 h), the platform was removed and a 60 s probe trial was executed. Following probe testing, visual cues were removed and mice were given 4 trials of Visible Platform Training (VPT) to assess for any gross sensorimotor or visual impairments. The escape latency, distance moved, duration in zones and velocity of each mouse was recorded using the Ethovision XT tracking system. The total time and percent time each subject spent in the quadrant previously containing the platform were determined as measures of platform location retention. Testing was conducted during the 9th week of the three-month dosing period. Mice that spent 100% of the time in non-target quadrants during the probe trial were excluded from data analysis; three Tg-V mice and one Tg-C31 mouse were excluded.

#### Trace fear conditioning (FC)

The Coulbourn Instruments (Whitehall, PA) fear conditioning system and FreezeFrame software were used for data acquisition and analysis. The protocol consisted of 1 day of Training, 1 day of contextual testing and 1 day of cued testing. The training and contextual test chambers were identical with identical contextual cues; the walls were made of aluminum, the floor of the chamber was a gray metal grid through which the US was delivered, light housing was yellow in color and the chambers were scented with mint extract. The chambers were cleaned with 10% Simple Green Solution (Sunshine Maker’s Inc, Huntington Beach, CA) between each mouse. Testing was conducted in a room with dim red color lighting. Cued Testing chambers were circular-shaped, made of plastic, light housing was blue in color, and scented with vanilla extract. These chambers were located in a room distinct from the training chamber with dim yellow color lighting and a white noise generator. The chambers were cleaned with 70% ethanol between each mouse. Both chambers were mounted within specially designed sound attenuating boxes. Each chamber had speakers mounted on the wall and included an exhaust fan and camera. On Day 1 training, each mouse was placed in the training chamber for 200 s. A tone (20 s, 80 dB, 2 kHz) was presented to the mouse followed by an electrical shock (intensity 0.5 mA, 2 s duration) 18 s after the end of the tone. This procedure was repeated 3 times with 60 s interval at the end of the shock until the next tone. The mouse was removed from chamber and returned to the home cage 60 s after the last shock. On Day 2, the mouse was returned into the training chamber without any tone or shock for contextual memory testing for 5 min. On Day 3, mice were place into the Cued Testing chambers with tones only (20 s, 80 dB, 2 kHz) presented 3 times with 60 s intervals after a 200 s habituation period. For each testing day session, percent time freezing was recorded. Mice received tones and shock pairings only on the training day. An overhead camera was used to record freezing behavior which was analyzed using FreezeFrame software. Fear conditioning studies were conducted during the 11th week of the three-month dosing period after all other behavioral testing had been completed. Based on the Grubb’s outlier testing, one Tg-V and one Ntg-C31 mouse were excluded from analysis.

### Statistics

All survival, morphological and behavioral studies were carried out in a blinded manner. Data were analyzed for statistical significance using GraphPad Prism software (version 8.0). Each study was completed with the listed number of samples, mice and measurements included in the figure legends. Data were assessed for normal distribution and parametric or non-parametric tests were applied accordingly. Specific statistical methods for each study, including ANOVA and post hoc analyses, are specified in the figure legends. For all cases, two-tailed tests were applied and a significance threshold was set at a p value of < 0.05. Graphical data are represented as bars indicating the data mean with error bars showing standard errors. For non-behavioral studies, data values beyond two standard deviations from the mean were considered outliers as a pre-hoc rule and removed. For behavioral studies, Grubb’s outlier analysis was applied as per standard, pre-hoc protocol for the Stanford Behavioral and Functional Neuroscience Laboratory, and cases of removed mice are indicated in the Results.

## Results

### LM11A-31 inhibits calpain activity and activation of cdk5, JNK and cofilin but not GSK3β in PS19 mice

**Fig. 1:**
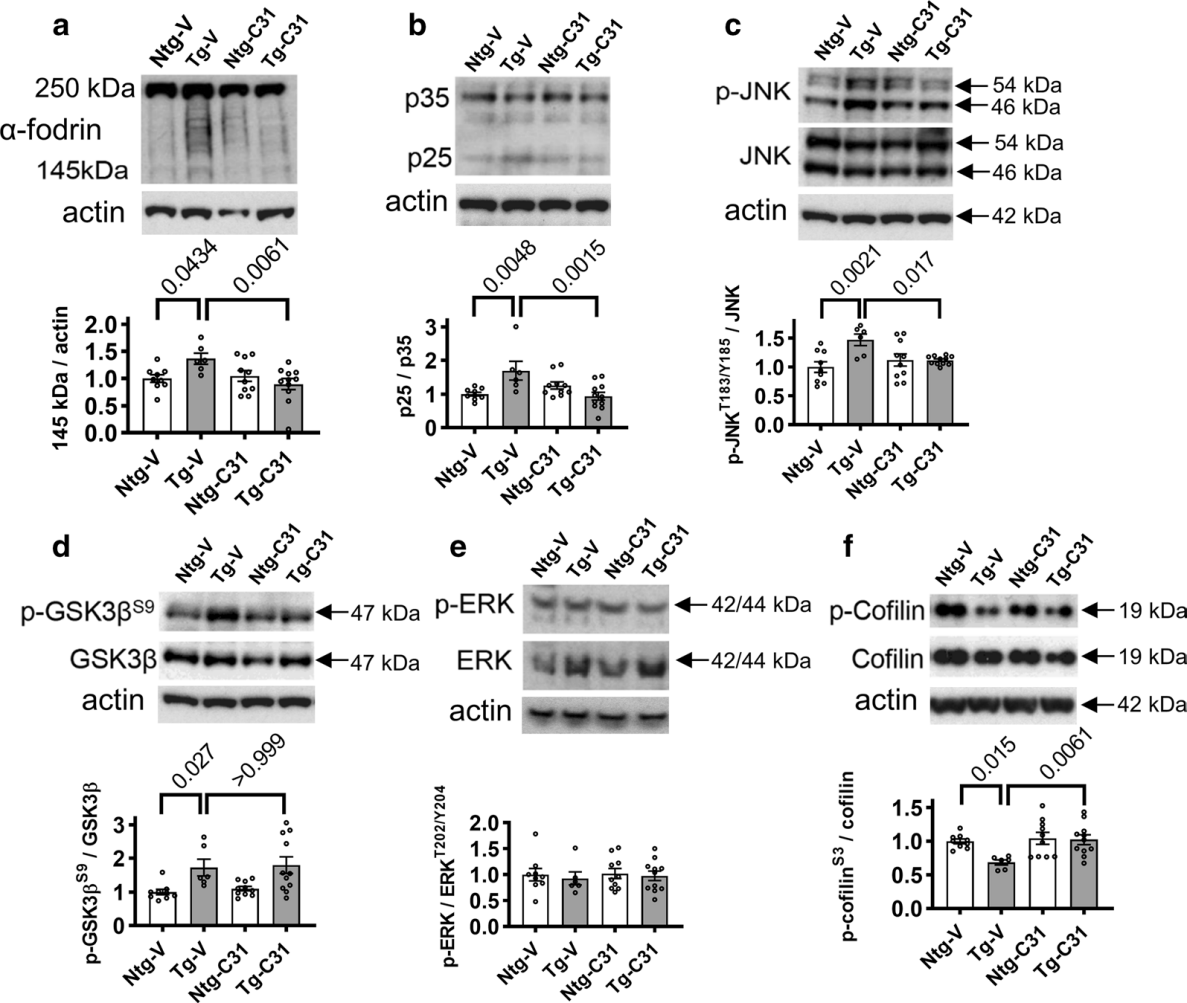
LM11A-31 treatment modulates mechanisms regulating tau cleavage and phosphorylation. **a**–**f** Western blots of hippocampal extracts (representative examples shown) were quantitated by determining ratios of cleaved to non-cleaved fragments or actin, or ratios of phospho (p)-protein over total protein and normalized to the mean of Ntg-vehicle mice **a** ratio of ~ 145 kDa α-fodrin calpain cleavage fragments to actin. **b** Ratio of CDK5 regulatory subunit p25 (active, calpain cleavage product) to full length p35 inactive subunit. **c** p-JNK^T183/Y185^ to total JNK. **d** p-GSK3β^S9^ to total GSK3β, **e** p-ERK^T202/Y204^ to total ERK. **f** p-cofilin^S3^ to total cofilin. p-values for the indicated comparisons are shown and statistical significance was determined using an ANOVA with post hoc Sidak’s multiple comparisons test; n = 6–11 mice per group, with two or three independent western blots averaged per animal

Evidence suggests that calpains can promote tau fragmentation in AD and other disease contexts [25] and p75^NTR^ signaling can regulate calpain activity [50]. Activation of calpains results in cleavage of the 250 kDa cytoskeletal protein α-fodrin to fragments of ~ 145 kDa, and the ratio of the cleaved fragments to actin serves as a measure of calpain activity. In 9-month old brain tissue samples, calpain activation was increased in Tg mice (p = 0.0434) consistent with previous observations in PS19 mice [81] and treatment with LM11A-31 decreased calpain activity (p = 0.0061) (Fig. 1a). Increased calpain activity also leads to the cleavage of the p35 regulatory subunit of cdk5 to the p25 constitutively active form, thereby promoting excessive cdk5 activity, and the ratio of p25 to p35 forms is a measure of cdk5 activation [27]. In 9-month old transgenic vehicle-treated tissue samples there was an increase in the ratio of p25 to p35 subunits (p = 0.0048) (Fig. 1b) consistent with elevated cdk5 activation in PS19 mice as has been previously reported [64] and with the upstream activation of calpain as noted above. Treatment with LM11A-31 prevented cdk5 activation (p = 0.0015), consistent with inhibition of calpain activity. p75^NTR^ signaling is also known to modulate JNK activation [22, 61] which can be assessed by measuring its phosphorylation at Thr183/185. p-JNK^Thr183/Tyr185^/total JNK levels were found to be elevated in PS19 mice (p = 0.0021) as reported in an earlier study [80] and this increase was prevented by LM11A-31(p = 0.017) (Fig. 1c).

LM11A-31 has been shown to promote the ability of p75^NTR^ signaling to activate AKT [47] and activation of AKT leads to inhibition of GSK3β [13] and JNK [41]. A trend for increased activation of GSK3β has been reported in PS19 mice [2]. In the present study, increased activation of GSK3β as determined by Ser9 dephosphorylation was detected in PS19 mice (p = 0.027), although treatment with LM11A-31 did not mitigate this increase (Fig. 1d). ERK activity has been implicated as a target of mutant tau toxicity [49], as well as a driver of tau pathology, though this remains unclear [56]. In the model utilized here, activation of ERK (phosphorylation at ERK^Thr202/Tyr204^) showed no difference between Ntg and PS19 mice and there was no effect of LM11A-31 (Fig. 1e).

p75^NTR^ modulates RhoA GTPase activation [43] which regulates cofilin phosphorylation and thereby influences actin cytoskeleton dynamics and synaptic spine stability [35]. Excess activation of RhoA promotes dephosphorylation of the cofilin Ser3 site which upregulates its activity and triggers synaptic spine collapse. Increased activation of cofilin is found in Alzheimer’s disease brain tissue [53], AD mouse models [77] and in PS19 mice [76]. AAV-mediated delivery of pseudo-active Ser3A-cofilin into the hippocampus of WT mice promotes tauopathy-related mechanisms including increased AT8 tau phosphorylation and degeneration of synapses, while crossing P301S tauopathy mice with cofilin+/− mice ameliorates this pathology [76]. In the present study, we detected a decrease in cofilin phosphorylation at the Ser3 site (increase in activated cofilin) in Tg mice (p = 0.015), consistent with earlier PS19 studies. Treatment with LM11A-31 resulted in a normalization of cofilin Ser3 phosphorylation (p = 0.0061) (Fig. 1f) and had no effect in Ntg mice.

### LM11A-31 treatment inhibits excess tau phosphorylation, acetylation, misfolding, fragmentation, accumulation of human mutant tau aggregates and accumulation of paired helical filaments

**Fig. 2:**
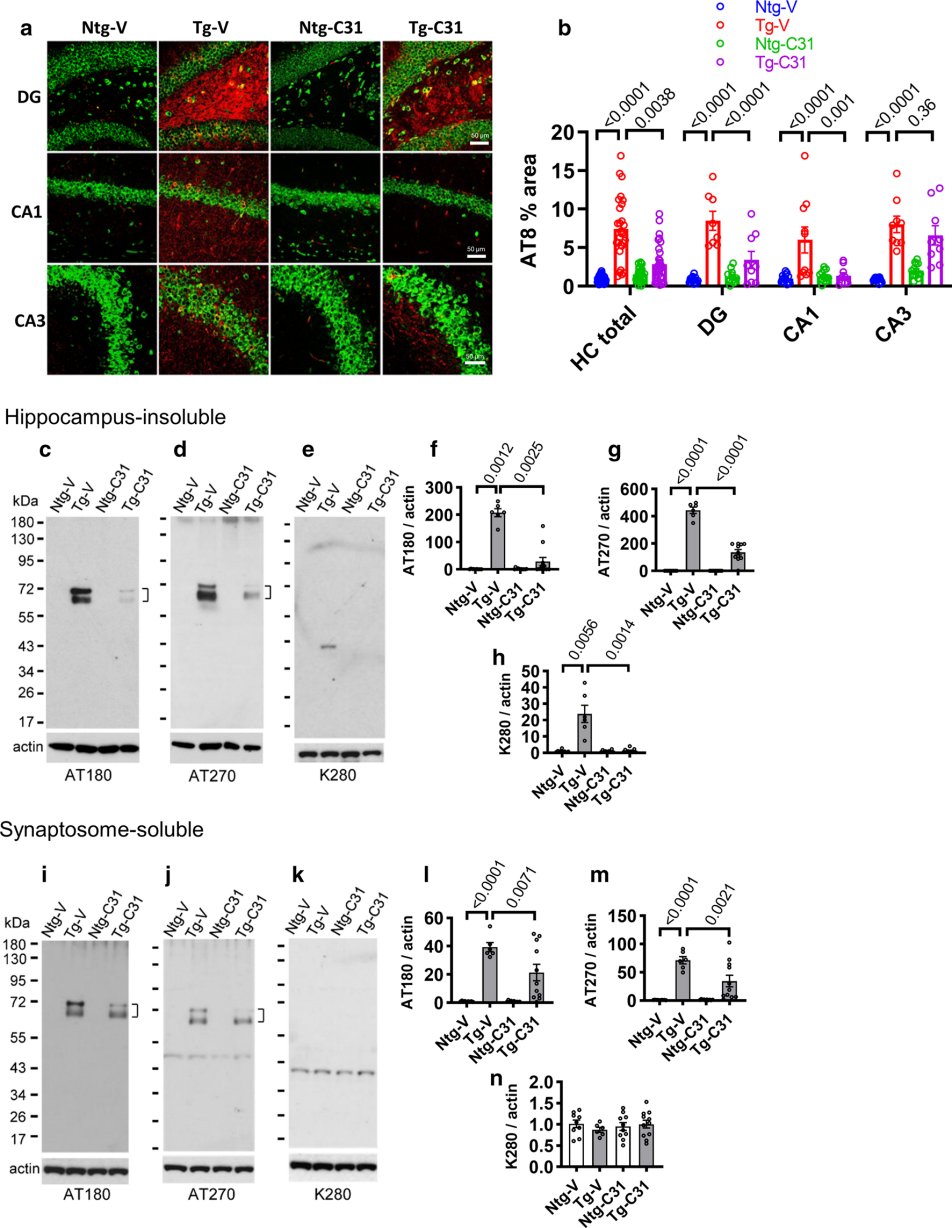
LM11A-31 reduces AT8 tau pathology, tau phosphorylation and tau^K280^ acetylation. **a** AT8 immunostaining in 9-month old untreated and treated Ntg and Tg mice (AT8, red; Nissl staining, green). Scale bar, 50 µm. **b** % of hippocampal area with AT8-positive immunostaining in post-treatment mice. For the total hippocampal region (HC total), significance determined using Kruskal–Wallis with Dunn’s post hoc multiple comparisons test. p values for the indicated comparisons are shown. For HC regions, ANOVA with Sidak’s post hoc multiple comparison testing; n = 8–13 mice per group. **c**–**e** Western blot analyses of sarkosyl-insoluble hippocampal lysates performed with the indicated antibodies. Primary p-tau bands of ~ 68 and 64 kDa (AT180, AT270) and an acetylated band (tau^K280^) at ~ 40 kDa appear in Tg samples. **f**–**h** Densitometric quantitation of the combined two primary p-tau bands and the single tau^K280^ band with p-values indicated. Statistical significance was determined using Kruskal–Wallis with post hoc Dunn’s testing (**f, h**) and ANOVA with post hoc Sidak’s testing (**g**); n = 6–11 mice per group with two independent western analyses averaged per animal. **i**–**k** Western blot analysis of soluble hippocampal synaptosome lysates performed with the indicated antibodies. p-Tau ~ 68/64 kDa (**i**, **j**) and acetyl-tau^K280^ ~ 40 kDa (**k**) bands are detected. Bands at ~ 45 kDa in all four lanes (**j**) are due to prior probing of blots with actin antibody and persistence of signal. **l**–**n** Densitometric quantitation of combined ~ 68/64 kDa p-tau or ~ 40 kDa acetyl-tau^K280^ signal with p-values for the indicated comparisons shown. Statistical significance was determined using ANOVA with post hoc Sidak’s multiple comparison testing; n = 6–11 mice per group, with two independent western blots averaged per animal

Since modulation of p75^NTR^ inhibited over-activation of multiple tau kinases (cdk5, JNK), and excess calpain activity and cofilin activation (relevant to AT8 epitope phosphorylation) in PS19 mice, it was of interest to determine whether LM11A-31 treatment would also be associated with reductions of tau molecular pathology including aberrant phosphorylation and acetylation, misfolding, fragmentation and aggregation. Immunostaining of hippocampal sections from age 9-month Tg mice with AT8 antibody (pSer202/Thr205) demonstrated increased signal in the hippocampus overall (p < 0.0001) with administration of LM11A-31 leading to a significant decrease (p = 0.0038) (Fig. 2a, b). In regional analyses, increases in Tg mice were found in dentate gyrus, CA1 and CA3 (p < 0.0001) and treatment was associated with a reduction of signal in the dentate (p < 0.0001) and CA1 (p = 0.001) areas, with a negligible reduction in CA3 (p = 0.36). Decreased AT8 signal as a result of treatment was also found in the cortex (p = 0.0521) (Additional file 1: Fig. S1).

Tau phospho-epitopes AT180 (pThr231) and AT270 (pThr181) were assessed using western blot analysis of sarkosyl-insoluble fractions [58]. Signals were nominal in extracts from Ntg mice from antibody AT180 (which recognizes human and rat tau) (p = 0.0012), and AT270 (which recognizes human tau) (p < 0.0001), while clearly prominent in human tau-expressing Tg mice. Two primary bands were observed in the 60–70 kDa range, at ~ 64 and ~ 68 kDa (Fig. 2c, d), a size range known to contain extensively studied pathological forms of tau including monomers with excess phosphorylation, oligomers of cleaved tau and filamentous species [12, 26, 33, 58], commonly associated with ~ 64 or ~ 68 kDa bands. Treatment with LM11A-31 was associated with significant reductions in the 60–70 kDa signal as measured by both phospho-epitope antibodies (AT180, p = 0.0025, AT270, p < 0.0001) (Fig. 2c, d, f, g). Since tau phosphorylation and acetylation states can each influence the other, and tau acetylation can promote its aggregation and contribute to synaptic impairment and degeneration, it was of interest to determine acetylation at the tau K280 site, which occurs in tauopathies and potentially contributes to fibril formation [69, 70]. Western blot signal was again nominal in sarkosyl-insoluble Ntg samples (p = 0.0056), indicating antibody specificity, but was abundant in PS19 Tg extracts at a MW of ~ 40 kDa (Fig. 2e, h), a size range similar to ~ 38 and ~ 43 kDa tau species reported to be associated with tau acetylation occurring with tau fragmentation [8]. Administration of LM11A-31 led to a marked reduction in signal to near trace levels (p < 0.0014) (Fig. 2h).

Given the likely important role of pathological tau in synapses [31, 32], western blot studies were conducted with soluble synaptosome fractions. Synaptosomal enrichment was confirmed by western blotting ([74, 75]) (Additional file 2: Fig. S2). A similar pattern was observed, with elevations of AT180 and AT270 signal in Tg mice (p < 0.0001 for each) and marked decreases associated with treatment (p = 0.0071 and 0.0021) (Fig. 2i, j, l, m). Interestingly, an increase in K280 acetylation was not detected in PS19 Tg soluble synaptosome fractions (Fig. 2k, n), perhaps indicating an accumulation of acetylated tau in sarkosyl-insoluble relative to soluble fractions and suggesting an association between acetylation and tau solubility.

**Fig. 3:**
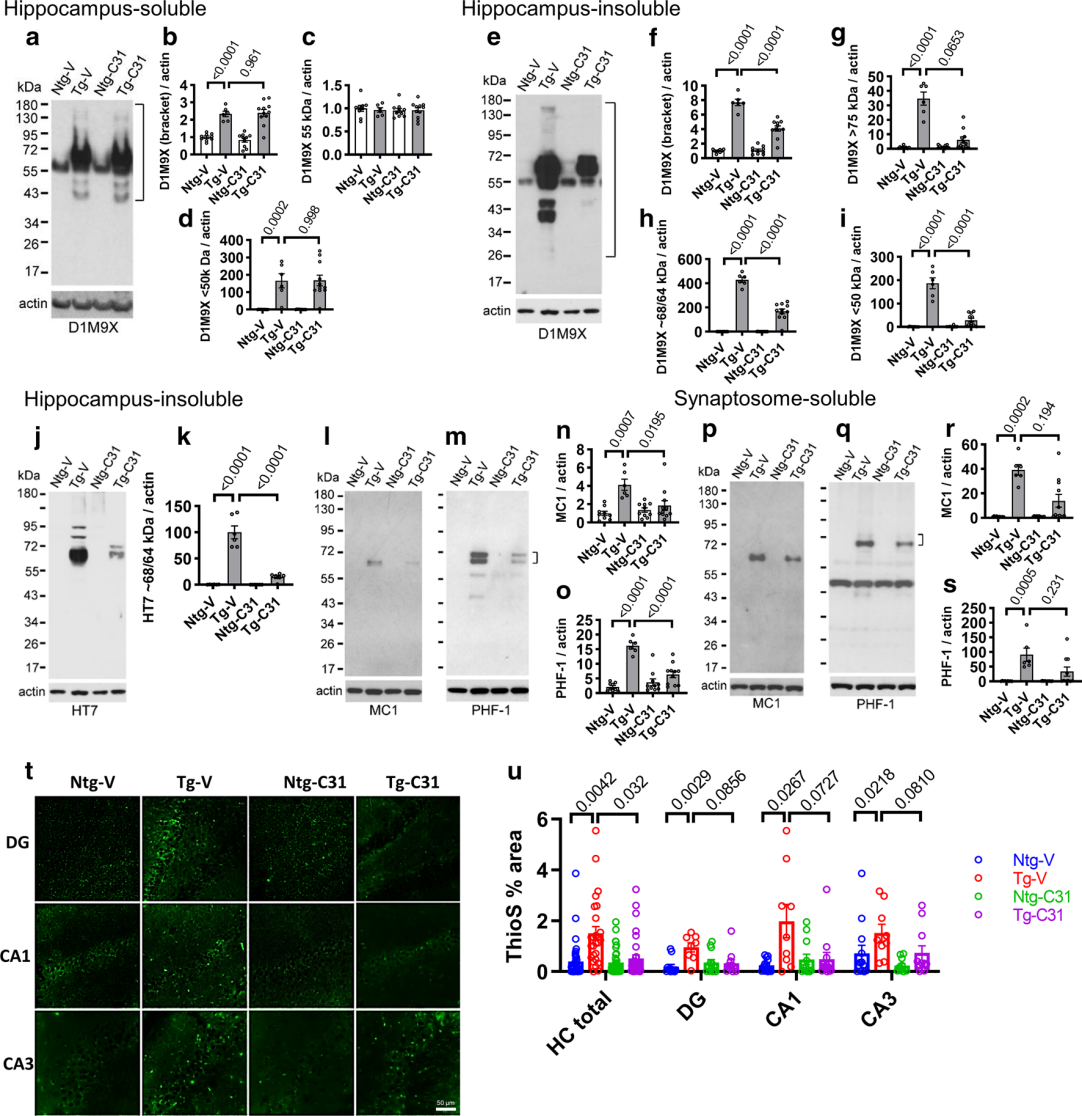
LM11A-31 reduces tau misfolding and aggregation. **a** Western analyses of soluble hippocampal lysates performed with tau D1M9X antibody for total tau. **b**–**d** Densitometric quantitation of bands in the indicated size ranges with p-values for the indicated comparisons shown. Statistical significance was determined using ANOVA with post hoc Sidak’s multiple comparison testing; n = 6–11 mice per group with two independent western analyses averaged per animal. **e** Western blot analysis of sarkosyl-insoluble lysates with D1M9X antibody. Over-exposed image demonstrates signal at < 50 and > 75 kDa, as well as at ~ 55 and ~ 68/64 kDa. Lower exposures were used for quantitation of ~ 68/64 kDa bands (Additional file 3: Fig. S3). **f**–**i** Densitometric quantitation of bands at the indicated size ranges with p-values indicated. Statistical significance was determined using ANOVA with post hoc Sidak’s testing (**f, h, i**) or Kruskal–Wallis with post hoc Dunn’s testing multiple comparison testing (**g**); n = 6–11 mice per group with two independent western analyses averaged per animal. **j** Western blot analysis of hippocampal sarkosyl-insoluble lysates performed with HT7 antibody specific to human total tau. Over-exposed image demonstrates signal at > 75 kDa. Lower exposures were used for quantitation of ~ 68/64 kDa bands (Additional file 3: Fig. S3). **k** Densitometric analysis of the ~ 64 kDa signal with p-values indicated. Statistical significance was determined using ANOVA with post hoc Sidak’s multiple comparison testing; n = 6 mice per group with two independent western analyses averaged per animal. **l**, **m** Western blot analyses of hippocampal sarkosyl-insoluble lysates performed with the indicated antibodies. **n**, **o** Densitometric analysis of immunoblots (MC1, ~ 64 kDa; PHF-1, 68/64 kDa bands combined) with p-values indicated. Statistical significance was determined using Kruskal–Wallis with post hoc Dunn’s testing (**n**) or ANOVA with Sidak’s post hoc multiple comparison testing **(o**); n = 6–11 mice per group, with two independent western blots averaged per animal. **p**, **q** Western blot analyses of soluble hippocampal synaptosome lysates performed with the indicated antibodies. Bands at ~ 45 kDa in all four lanes (**q**) are due to prior probing of blots with actin antibody and persistence of signal. **r**, **s** Densitometric analysis of immunoblots blots (~ 64 kDa for MC1 or ~ 68 kDa for PHF-1) with p-values indicated. Statistical significance was determined using Kruskal–Wallis with post hoc Dunn’s testing; n = 6–11 mice per group with two independent western blots average per mouse. **t** ThioS hippocampal staining in 9-month old mice. **u** Quantification of hippocampal ThioS-positive staining. Statistical significance was determined using Kruskal–Wallis with post hoc Dunn’s testing; n = 8–13 mice per group

Since LM11A-31 inhibited excess phosphorylation at three tau epitopes, along with inhibiting K280 acetylation, we posited that it would also reduce tau misfolding and aggregation. Assessment of soluble hippocampal extracts with the D1M9X total tau antibody, which recognizes both mouse and human tau, demonstrated a primary band in the range of 50–60 kDa consistent with tau monomers, along with > 60 and < 50 kDa bands in Tg extracts (Fig. 3a) characteristic of oligomers [24] and cleaved tau fragments [51], respectively. Levels of oligomers and cleaved fragments were markedly increased in Tg mice while essentially undetectable in Ntg mice, consistent with antibody specificity for these multiple aberrant tau species. Treatment with LM11A-31 had no detectable effects on levels of signal in the tau physiological monomer range and no detectable effects on levels of other soluble tau species detected by the D1M9X antibody (Fig. 3b–d). Assessment of sarkosyl-insoluble fractions with the D1M9X antibody demonstrated a ~ 55 kDa band consistent with physiological tau monomer in Tg and Ntg mice (Fig. 3e). Additional bands detected in Tg mice were quantitated in combination, and separately in the size range categories of < 50, 60–70 and > 75 kDa. Overall signal (Fig. 3f) and signal in each of the three ranges (Fig. 3g–i) was markedly elevated in Tg mice (each p < 0.0001) consistent with a wide range of pathological tau species. Absence of signal in Ntg extracts suggested specificity of the antibody in the insoluble extracts. In the < 50 and ~ 68/64 kDa ranges signal was significantly decreased by LM11A-31 treatment (each p < 0.0001), and in the > 75 kDa range there was a strong trend towards a decrease (p = 0.0653). These findings suggested that administration of LM11A-31 was associated with decreased accumulation of oligomeric (> 75 kDa), potentially fibrillary and other oligomeric (~ 68/64 kDa) as well as cleaved forms (< 50 kDa) of insoluble tau.

Given the potential confounds of the D1M9X antibody detection of both mouse and human total tau, the HT7 total tau antibody which detects human but not mouse tau was used to verify the effects of treatment on human mutant forms of insoluble aggregated tau. Western blots with HT7 demonstrated abundant signal at ~ 68/64 kDa as well as higher molecular weight bands consistent with other studies [82] with trace to absent levels in Ntg mice indicating antibody specificity (Fig. 3j). Treatment was associated with a substantial decrease in each of these bands with the higher molecular weight bands reduced to trace levels. Quantitation of the ~ 68/64 kDa signal demonstrated a significant reduction in association with treatment (p < 0.0001) (Fig. 3k).

Analysis of sarkosyl-insoluble fractions with the MC1 antibody for detection of tau misfolding, an early event in pathological tau formation leading to tau aggregation, detected no signal in Ntg mice but an abundant ~ 64 kDa signal in Tg samples (p = 0.007) (Fig. 3l). Exposure to LM11A-31 resulted in reduced MC1 signal (p = 0.0195) (Fig. 3n). To further examine effects on a range of pathogenic species, a later, intermediate-stage form of pathological tau, PHF-tau, was assessed through western blot analysis of sarkosyl-insoluble fractions using paired-helical filament (PHF-1) antibody (pSer396/Ser404, specific to human tau). Signal was undetectable in Ntg mice but readily apparent in PS19 mice primarily as ~ 64 and ~ 68 kDa bands (p < 0.0001) and modulation of p75^NTR^ resulted in a marked reduction in both bands (p < 0.0001) (Fig. 3m, o).

Analysis of soluble synaptosome fractions revealed similar patterns. MC1 signal was nominal in Ntg extract but was present in Tg samples (p = 0.0002). Exposure to LM11A-31 led to an apparent reduction in synaptosomal misfolded tau which did not reach significance (p = 0.194) (Fig. 3 p, r). Similarly, PHF-1 measurements in soluble synaptosome fractions demonstrated abundant ~ 64 kDa signal in PS19 Tg fractions (p = 0.0005) and a trend towards reduction in fractions from treated PS19 Tg mice (p = 0.231) (Fig. 3q, s).

Finally, effects on mature forms of pathological tau, including tau paired helical filaments, were examined using Thioflavin S staining of hippocampal tissue. Signal within the hippocampus overall was increased (p = 0.0042) consistent with earlier PS19 mouse studies [15]. Administration of LM11A-31 was associated with significantly reduced staining (p = 0.032) (Fig. 3t, u). Levels within DG, CA1 and CA3 regions were increased in PS19 mice (p = 0.0029; p = 0.0267; p = 0.0218) and treatment with LM11A-31 was associated with trends for decreased signal in the individual DG, CA1 and CA3 regions (p = 0.0856; p = 0.0727; p = 0.0810).

While decreases in tau phosphorylation, misfolding and aggregation are consistent with the inhibition of the pathological activation of multiple tau kinases, it is also possible that increases in levels of tau phosphatase [57] or autophagy [62] might contribute to the tau aggregate lowering effect of LM11A-31. PP2A, considered the primary tau phosphatase [57], showed no changes in levels in western blot analyses in Tg vs Ntg mice or as a result of drug treatment. Similarly, no changes in levels of the p62 autophagy marker, a measure used in tauopathy targeting models, were detected in Tg vs Ntg mice or in association with drug treatment (Additional file 4: Fig. S4).

### LM11A-31 treatment inhibits accumulation of seed-competent tau

**Fig. 4:**
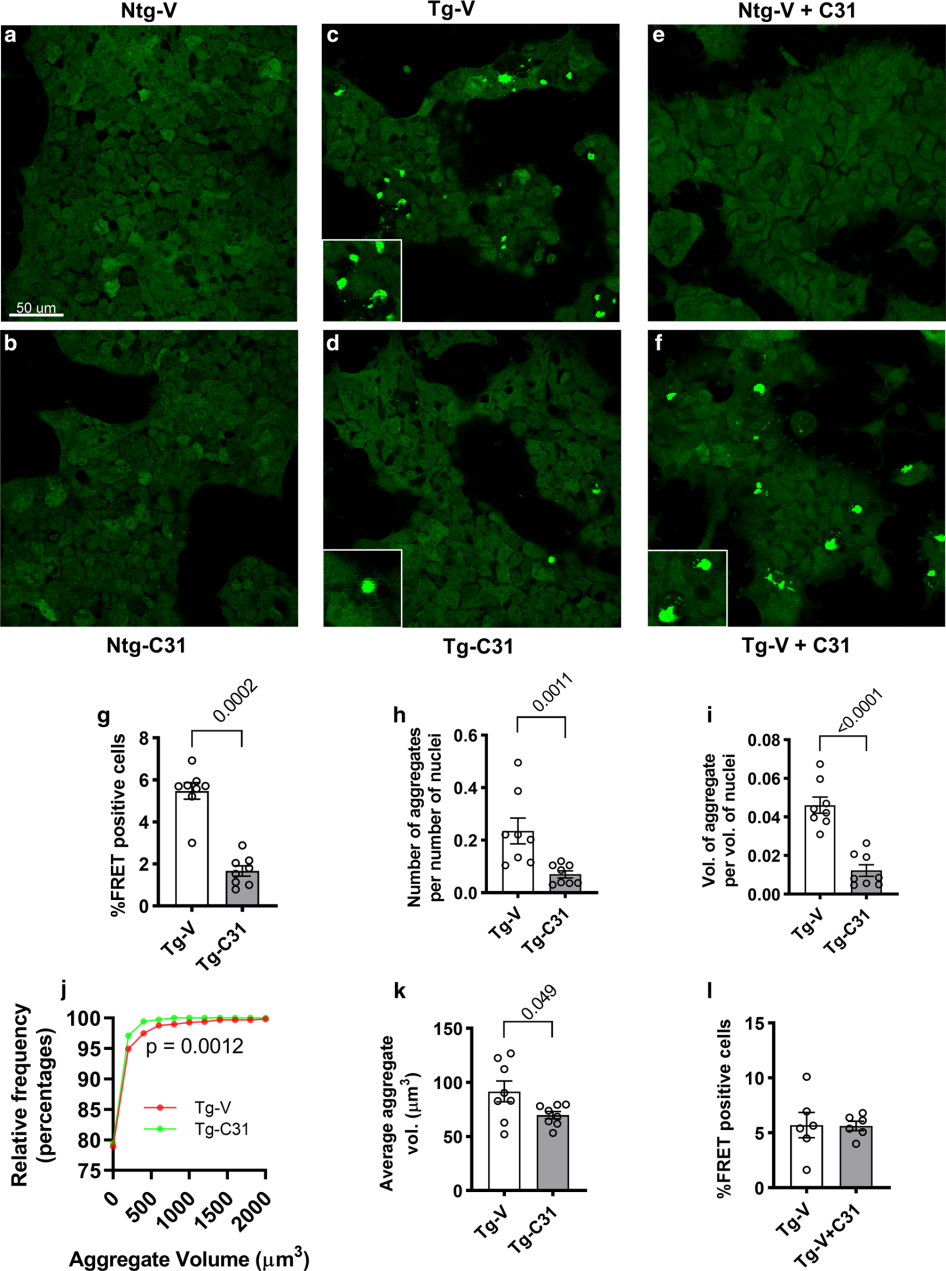
LM11A-31 treatment reduces tau seeding activity. **a**–**d** Confocal FRET images of cultured HEK 293T RD-P301S-CFP/-YFP biosensor cells transfected with hippocampal homogenate from 9-month old vehicle- (**a**, **c**) or LM11A-31- treated (**b**, **d**) Ntg and Tg mice. **e**, **f** FRET images of biosensor cells transfected with brain lysates from 9-month old vehicle-treated Ntg (**e**) and Tg (**f**) mice in the presence (**e**, **f)** of LM11A-31 in the bioassay test medium. In Tg brain lysate transfected conditions, abundant FRET-positive aggregates are present with a notable reduction associated with lysate from LM11A-31 treated mice. Scale bar: 50 µm (inserts, 3X). **g**–**k** Quantitative analysis of FRET-positive aggregates. **g** Percentage of FRET-positive cells. **h** Number of aggregates per number of nuclei. **i** Volume of aggregates per volume of nuclei. **j** Cumulative frequency plot of aggregate volumes. **k** Average aggregate volume. Statistical significance was determined using Mann–Whitney (**g**, **h**), student t (**i**, **k**) and two-sample Kolmogorov–Smirnov testing (**j**). For each above outcome measure, n = 8 wells assessed from 3 independent studies with 2 wells per mouse and 4 mice per group. **l** Acute addition of LM11A-31 (100 nM) during transfection with vehicle-treated Tg lysate had no detectable effect in reducing FRET signal (student t test, n = 6 z-stacks)

Tau prion-like seeding activity has been postulated to play a key role in AD and other tauopathies [9, 16, 30, 73]. Tau seeding has been associated with sarkosyl-insoluble aggregates [18] and 64 kDa-positive filamentous species [33]. The ability of LM11A-31 to affect multiple forms of tau modifications and reduce accumulation of many of these tau species suggested that it might inhibit the formation/accumulation of seed-competent tau. Tau seeding activity can be measured by the addition of brain tissue extracts to HEK-293 cells expressing the tau P301S monomeric repeat domain (TauRD) fused with either cyan (TauRD-CFP) or yellow fluorescent protein (TauRD-YFP) [30], with uptake of seed-inducing tau species triggering intracellular tau misfolding and aggregation, resulting in a Forster Resonance Energy Transfer (FRET) signal. Hippocampal tissue lysates derived from Ntg or Tg mice after the three-month treatment period were applied to the FRET biosensor assay. Tissue was harvested from mice 24 h after the last drug dose, a time point at which drug levels would be trace or undetectable [38]. Addition of Ntg extract resulted in no detectable FRET-positive inclusions while the addition of extract from vehicle treated Tg mice triggered abundant FRET-positive aggregates (Fig. 4a**-d**). Application of extract from LM11A-31-treated Tg mice demonstrated substantial reductions in the number of % FRET-positive cells (p = 0.0002); and the number (p = 0.0011) and total volume (p < 0.0001) and a significant left-shift of the volume cumulative frequency curve (p = 0.0012) of FRET-positive aggregates (Fig. 4g–j). Average aggregate size was modestly but significantly (p = 0.049) reduced as well (Fig. 4k). Addition of LM11A-31 at a concentration of 100 nM, a level typically evoking maximal responses in in vitro studies, to the FRET biosensor assay in the presence of Ntg extract from vehicle-treated mice did not promote FRET signal (Fig. 4e). Finally, addition of LM11A-31 in the presence of Tg extract from vehicle-treated Tg mice led to no decrease in FRET-positive aggregates, indicating that drug effects on seeding were due to engagement of mechanisms in vivo rather than direct effects of residual free drug on the assay (Fig. 4f, l).

### LM11A-31 treatment decreases degeneration of synaptic spines and synapses

**Fig. 5:**
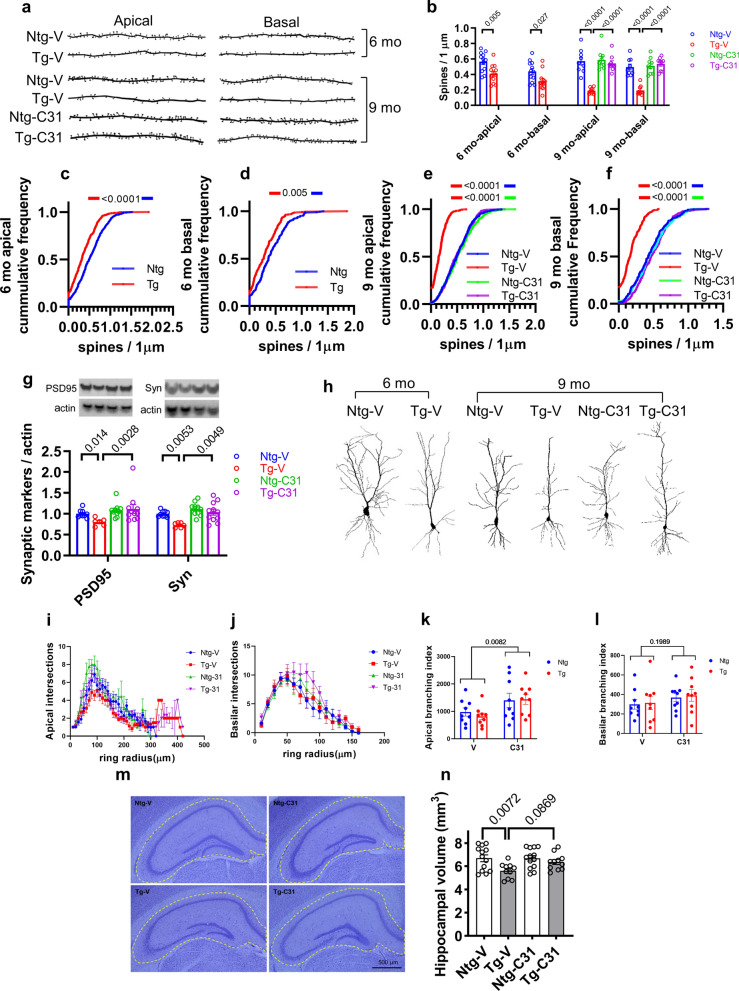
LM11A-31 treatment attenuates neuronal degeneration. **a** Images reconstructed from Neurolucida tracings of Golgi-stained hippocampal CA1 pyramidal neuron dendritic segments from 6- and 9-month-old mice. Scale bar, 50 µm. **b**–**f** Quantitative analyses of dendritic spine densities. For 6-month aged mice, three neurons/mouse with four mice/condition, n = 12 neurons from each group. For 9-month age mice, three neurons/mouse with three mice/condition, n = 9 neurons from each group. **b** Statistical significance was determined using student t testing for age 6 months and ANOVA with post hoc Sidak’s multiple comparisons testing for age 9 months, **c**–**f** Cumulative frequency distributions of dendritic spine density. Statistical significance was determined by pairwise two-sample Kolmogorov–Smirnov testing. *p* value comparisons are indicated. **g** Hippocampal synaptosomal protein extracts were assessed by western blots. The ratio of each synaptic protein to actin was determined; n = 6–11 mice, with four independent western blots averaged per animal. Statistical significance was determined using Kruskal–Wallis testing with post hoc Dunn’s multiple comparisons testing. **h** Images reconstructed from Neurolucida tracings of pyramidal neurons from 6- and 9-month old mice. Scale bar = 10 µm. **i**, **j** For neurite Sholl ring intersection analyses, numbers of neurons assessed were as follows: 6-month mice, three neurons/mouse with four mice/condition, n = 12 neurons from each group; 9-month mice, three neurons/mouse with three mice/condition, n = 9 neurons from each group. Statistical significance between groups was analyzed by three-way ANOVA (described in Results). **k**, **l** Branching index analysis of Sholl intersection data. Statistical significance was determined using two-way ANOVA, p-values are indicated. **m** Nissl-stained coronal sections with regions selected for hippocampal volume measurements outlined. **n** Hippocampal volume analysis. One-way ANOVA with Sidak’s post hoc multiple comparison testing, n = 10–13 mice per group

Accumulation of toxic tau aggregates is likely a key mechanism leading to degeneration of spines, synapses and neurites in human tauopathies as well as in tauopathy mouse models [31, 36, 46]. In the PS19 model, decreases in synaptic markers are evident by age 4 months [79]. Since LM11A-31 diminishes formation of tau aggregates and tau seeding activity, we tested the hypothesis that treatment might prevent or reverse loss of dendritic spine density. Counts of Golgi-stained spines in hippocampal pyramidal neurons at age 6 months demonstrated significant decreases in spine density in PS19 Tg vs Ntg apical and basal dendrites (29%, p = 0.005, and 27%, p = 0.0027, decreases, respectively) (Fig. 5a, b). Cumulative frequency analysis of spine density per dendrite segment of apical and basal dendrites further demonstrated a notable reduction in spine density (p < 0.0001 for apical spines; p = 0.005 for basal spines) (Fig. 5c, d) at the 6-month time point. Spine counts performed in vehicle-treated Tg mice at the age of 9 months, at the end of the 3-month treatment period, revealed an expected further decrease in spine density in untreated Tg apical and basal dendrites (55% and 50% decreased density, respectively, p < 0.0001 for each) (Fig. 5a, b). In Tg mice treated with LM11A-31, no difference in spine density between Tg and Ntg mice could be detected in either apical or basal spines, consistent with a highly significant drug effect (p < 0.0001). Cumulative frequency analysis demonstrated a broad and even drug response across a wide range of spine densities, overlapping the Ntg curves with increased maximum densities relative to the Tg curve and without clear subpopulation effects (Tg-V vs Tg-C31, p < 0.0001 for both basal and apical spines) (Fig. 5e, f). These findings indicated that treatment was associated not only with a prevention of further spine loss after the 6 mo of age treatment initiation, but a reversal of the spine loss that was present prior to the initiation of treatment. Western blot studies of synaptosome fractions using the post-synaptic marker, PSD-95, demonstrated a 25% decrease in hippocampal tissue samples in 9 months old Tg vs Ntg mice (p = 0.014) (Fig. 5g). In Tg mice treated with LM11A-31, drug exposure was associated with a significant increase in signal (p = 0.0028). A similar pattern was found using the presynaptic marker synaptophysin where a 34% decrease in signal was found in Tg samples (p = 0.0053), with again a significant reversal effect (p = 0.0049) associated with treatment (Fig. 5g). In summary, three independent markers of synaptic degeneration demonstrated notable responses to p75^NTR^ modulation.

### LM11A-31 promotes pyramidal neuron neurite complexity

Analysis of apical neurite Sholl ring intersections (Fig. 5l) using three-way ANOVA showed significant main effects of genotype (F(1, 1334) = 8.252, p = 0.0041) and treatment (F(1, 1334) = 45.93, p < 0.0001), without significant treatment/genotype interaction (F(41, 1334) = 1.691, p = 0.4369). Further inspection of the Sholl distributions indicated fewer intersections of transgenic apical neurites than wild type in the region ~ 100 µm from the cell body, with both genotypes increasing similarly with LM11A-31 treatment there, and a tendency for extension of transgenic neurites substantially beyond 300 µm not seen in the wild-type and not affected by drug treatment. Similarly, basilar neurites (Fig. 5j) demonstrated significant main effects of genotype (F(1, 576) = 4.693, p = 0.0307) and treatment (F(1, 576) = 4.930, p = 0.0268), but in addition showed a significant interaction between the two (F(1, 576) = 7.042 p = 0.0082), with the distributions suggesting a greater effect of LM11A-31 on transgenic neurites, which did not reach significance in post hoc testing, and nominal difference between the genotypes in the absence of the drug. Evaluation of the branching index developed by Garcia-Segura and Perez-Marquez [21] (Fig. 5k, l) which is weighted towards complexity arising at greater distances from the cell body, revealed a significant main effect of drug treatment (F(1, 32) = 7.956, p = 0.0082) in apical neurites without a significant genotype effect (F(1, 32) = 0.1029, p = 0.7505), while in basilar neurites no significant differences with treatment or genotype were detected for this parameter. Overall, these findings suggest small but significant differences between genotypes with tendency towards lesser apical complexity in transgenics, and a substantial increase in complexity mediated by LM11A-31 treatment affecting both genotypes.

### Hippocampal volume and structure

In clinical tauopathies and mouse models cellular-level changes are reflected in morphologic changes in larger structures, and measures of hippocampal volume have been used as indicators of neurodegeneration in tauopathy mouse models. PS19 mice demonstrate an age-dependent decrease in hippocampal volume that begins to become apparent by age 9 months [78, 79]. In the present study, volume measurements following the 3-month treatment course at age 9 months demonstrated a significant 18% decrease in volume in Tg comparted to Ntg mice (p = 0.0072). Within Tg mice, application of LM11A-31 vs vehicle resulted in a partial degree of protection from volume loss (p = 0.0869) (Fig. 5m, n).

### LM11A-31 decreases activation of microglia

**Fig. 6:**
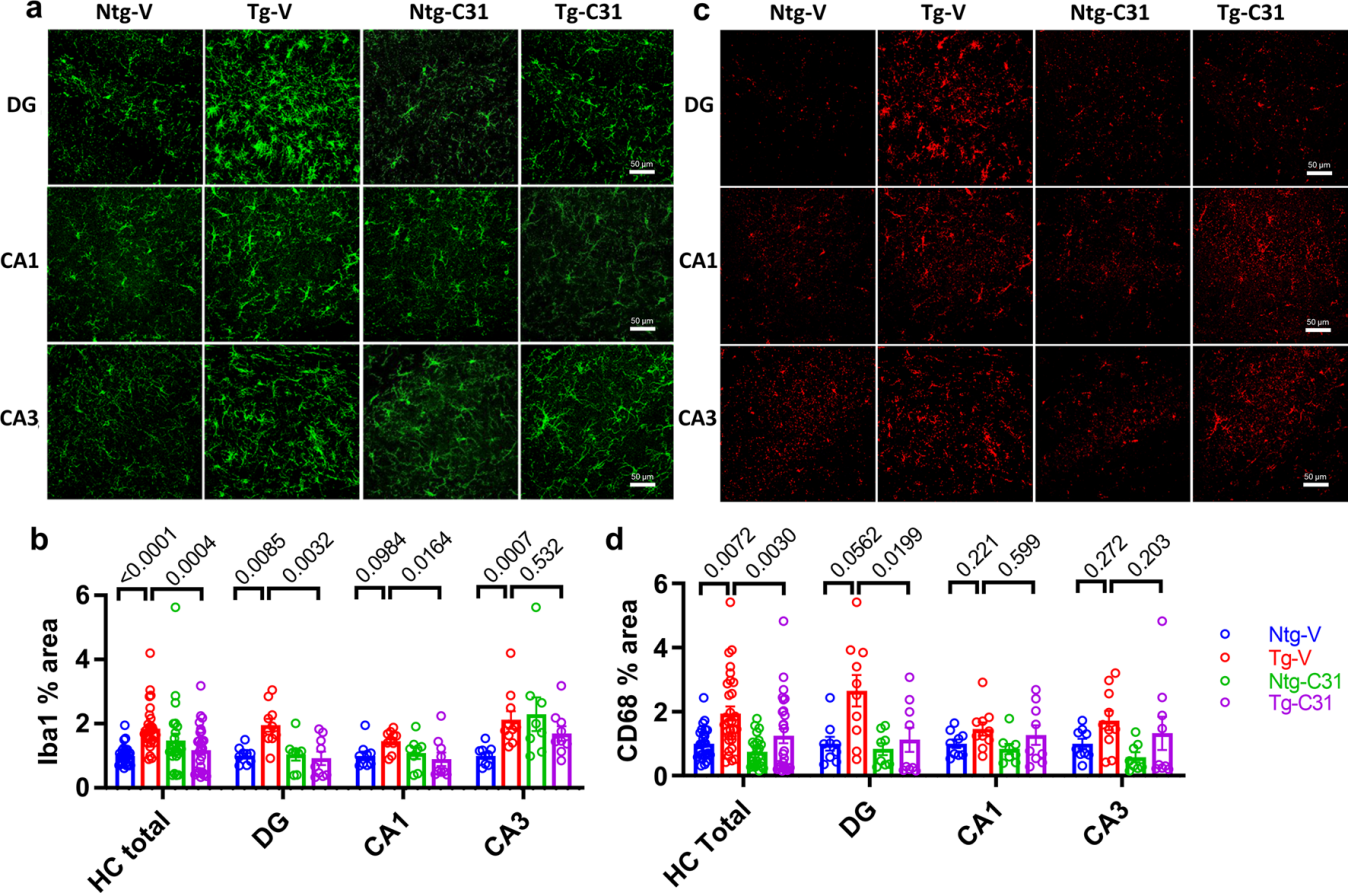
LM11A-31 treatment decreases activation of microglia. **a** Iba1 and **c** CD68 hippocampus immunostaining in 9-month old mice. Quantification of **b** Iba1- and **d** CD68-positive staining measured as % hippocampal area demonstrating signal. Statistical significance was determined using Kruskal–Wallis with post hoc Dunn’s testing; n = 8–10 mice per group, p-values are indicated

PS19 mice develop an age-dependent increase in microglial activation as reflected by expression of Iba1 and CD68 [44, 79]. In the present study, increased Iba1 signal was found in the hippocampus overall (p < 0.0001) and within the three regional areas of dentate gyrus (p = 0.0085), CA1 (p = 0.0984) and CA3 (p = 0.0007) (Fig. 6a, b). Administration of LM11A-31 caused a significant decrease in overall hippocampal signal (p = 0.0004) and in the dentate gyrus (p = 0.0032) and CA1 (p = 0.0164) regions with no effect detected in CA3. An increase in CD68 signal was present in the hippocampus overall (p = 0.0072) with trends of increased signal within individual regions; DG (p = 0.0562), without clear effects in CA1 and CA3 (Fig. 6c, d). LM11A-31 led to a significant mitigation of the increased CD68 signal in the combined hippocampal regions (p = 0.0030) and a significant decrease in the DG (p = 0.0199) but was again without clear effects in CA1 and CA3. Western blot analysis of GFAP levels demonstrated a significant increase in Tg vs Ntg mice (p = 0.015) and this increase was also not affected by treatment with LM11A-31 (Additional file 5: Fig. S5).

### LM11A-31 prolongs PS19 mouse survival

**Fig. 7:**
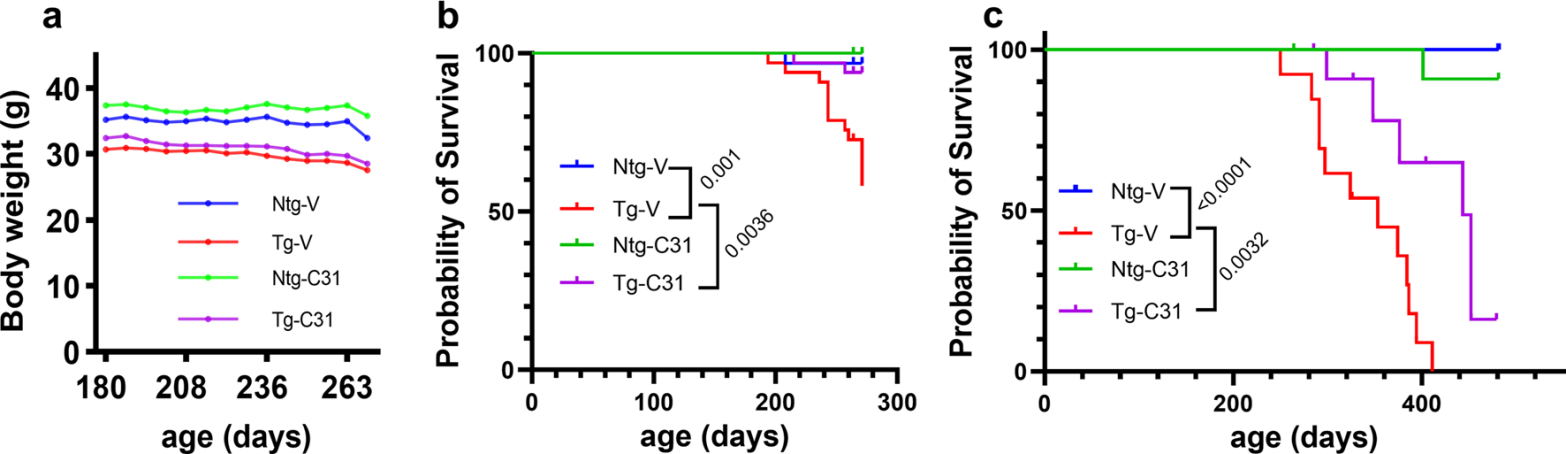
Effect of LM11A-31 treatment on survival. **a** Body weight monitoring demonstrates reduced weights in Tg mice. Statistical significance between groups was analyzed by mixed effects analysis. Group effect for genotype, p < 0.0001; n = 32–33 mice per group. **b** Survival analysis of cohorts treated 6–9 months with study terminated at 9 months of age. Data from two independent treatment cohorts is combined. LM11A-31 treated Tg mice demonstrated a reduced death rate compared to vehicle-treated Tg mice. Statistical significance was determined using the log-rank (Mantel-Cox) test; Ntg-V, n = 32–33 mice per group, p-value comparisons are indicated. **c** Survival analysis of mice with treatment starting at age 6 months, with termination of study at 481 days of age. Statistical significance was determined using the log-rank (Mantel-Cox) test; n = 12–13 mice per group, p-value comparisons are indicated

Similar to FTDP-17 subjects who carry a P301S tau mutation [6], PS19 mice demonstrate accelerated mortality and 80% of mice have been reported to die by 12 months of age [79]. Reduced body weights were found in Tg mice (p < 00001), as previously described [4] and administration of LM11A-31 had no significant effect (Fig. 7a). In two cohorts of mice undergoing treatment with vehicle or LM11A-31 from 6 to 9 months of age, survival rates were recorded. For these cohorts combined, survival rate for Tg-vehicle mice was 64% at the 9-month time point while rates for vehicle- and drug-treated Ntg mice were 97% and 100%, respectively (p = 0.001, Ntg-vehicle versus Tg-vehicle) (Fig. 7b). Treatment of Tg mice led to an improvement in survival rate to 94% at 9 months (survival analysis p = 0.0036). A separate trial was conducted in which survival was the primary endpoint (Fig. 7c). In the Tg-V group, 50% reached the endpoint by 327 days whereas in the Tg-drug group, 50% reached this endpoint at 404 days. One animal survived until the designated termination of the study at 481 days. Treatment with LM11A-31 extended survival in Tg mice with an increase in median survival time from 334 to 443 days (33% increase) (survival analysis p = 0.0032). Over the same time period, 100% and 96% of Ntg mice, treated with vehicle or drug respectively, survived.

### LM11A-31 improves hippocampal behavioral outcomes

**Fig. 8:**
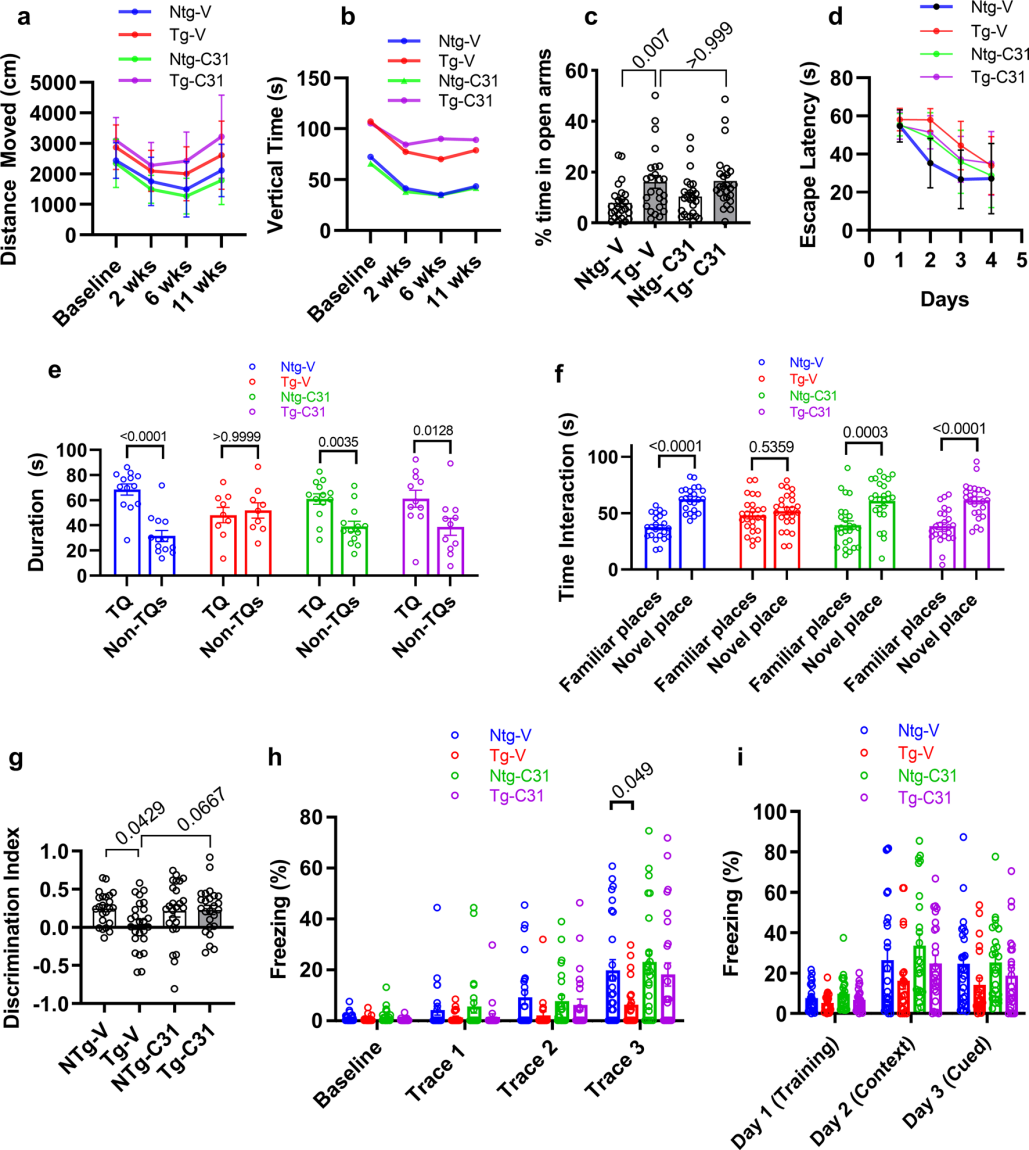
Effect of LM11A-31 treatment on behavior. **a** Distance moved in open field testing. Statistical significance between groups was analyzed by three-way ANOVA. Group effect for genotype, p < 0.0001; n = 22–26 mice per group. **b** Vertical time monitoring. Statistical significance between groups was analyzed by restricted maximum likelihood model. Group effect for genotype, p < 0.0001; n = 22-26 mice per group. **c** Elevated-plus maze analysis. Statistical significance was determined using Kruskal–Wallis with post hoc Dunn’s testing; n = 25 mice per group; p-values are indicated. **d** Morris Water Maze, learning phase assessed with escape latency assessment. Statistical significance between groups was analyzed by three-way ANOVA. Group effect for genotype, p = 0.0112; n = 11–13 mice per group. **e** Probe trial testing. Pairwise target versus non-target significance was determined with Mann–Whitney testing, p-values are indicated; n = 11–13 mice per group. **f** Novel place recognition testing. Familiar versus novel place recognition assessed using paired t testing, p values are indicated, n = 23–25 mice per group. **g** Novel place recognition discrimination index. Statistical significance was determined using ANOVA with post hoc Sidak’s multiple comparison testing; n = 23–25 mice per group. **h** Contextual fear conditioning, trace analysis. Statistical significance was determined using Kruskal–Wallis with post hoc Dunn’s testing; n = 23–25 mice per group. **i** Contextual and cued memory testing

Locomotor activity, was tested in an activity chamber at baseline prior to dosing and at three time points during the 3-month dosing period (Fig. 8a, b**)**. Open-field analysis showed significant main effects of genotype for distance moved (F(1, 94) = 37.14, p < 0.0001) and vertical rearing time (F(1, 97) = 41.34, p < 0.0001), with increases of both of these measures of activity in transgenic animals, with no significant effects of drug treatment. These findings were consistent with locomotor hyperactivity previously observed in PS19 mice [4, 68]. Prior behavioral assessment of PS19 mice also demonstrated reduced anxiety-like behavior [4, 68]. In the present study, monitoring of behavior in the Elevated Plus Maze confirmed an increase in time spent in open arms consistent with reduced anxiety in Tg mice (Fig. 8c, p = 0.007). Treatment with LM11A-31 also had no effect on this parameter.

PS19 mice exhibit deficits in spatial learning and memory as assessed by escape latency in the Morris Water Maze (MWM) [4]. In the present study, there was a significant increase in escape latency in Tg mice (main effect F(1, 42) = 7.038, p = 0.0112), in one of two cohorts tested and no effect of LM11A-31 treatment (Fig. 8d). By day 4, within the cohort demonstrating impaired learning by Tg-V mice, all four treatment groups demonstrated successful acquisition of the escape platform location (Fig. 8d). The cohort demonstrating no learning impairment was not further studied. In probe trials with the remaining cohort, Ntg mice, treated with either vehicle (p = 0.0001) or drug (p = 0.035) demonstrated a clear quadrant preference while Tg-V mice had no detectable preference for the target quadrant, suggesting memory impairment (Fig. 8e). In contrast, Tg mice treated with LM11A-31 demonstrated target quadrant preference (p = 0.0128). In Visible Platform Training, no deficits were detected in any of the four treatment groups indicating that the lack of demonstrated quadrant preference in Tg mice was not secondary to visual, motor or motivation deficits.

Spatial learning and memory in PS19 mice are also impaired in novel place recognition (NPR) and novel object recognition (NOR) tasks [68]. NPR testing after 7 weeks of vehicle or drug treatment showed deficits in Tg mice (Fig. 8f, g). Ntg mice showed the expected increased exploration time of an object in a novel location relative to an identical object in a known location, indicating successful memorization of object–place relationships (p < 0.0001). In contrast, in vehicle-treated Tg animal testing, exploration times of the object in the novel location did not differ significantly from those of the object in the known location, consistent with learning and memory impairment. LM11A-31-treated Tg mice, however, showed place discrimination ratios that were restored similar to those of Ntg mice (p < 0.0001) (Fig. 8f). Further direct comparisons using discrimination indexes showed similar deficits in Tg mice (p = 0.0429) with a strong trend towards correction by drug treatment (p = 0.0667). LM11A-31 did not improve performance in Ntg mice, thereby excluding a non-specific cognitive enhancing effect. Testing of NOR after 7 weeks of vehicle or drug treatment demonstrated no clear deficits in Tg mice, thereby providing no opportunity to assess the ability of LM11A-31 to reduce a deficit. The finding here that NPR, but not NOR, assessment detected decreased performance in Tg mice is consistent with prior studies suggesting relatively higher sensitivity of NRP versus NOR when both are included in one study [37].

PS19 mice have been reported to have deficits in fear-associated learning and memory appearing as early as age 5–6 months [68]. PS19 mice underwent fear conditioning followed by cued- or context-based retrieval testing. Relative to baseline measurements, both genotypes acquired the task equally well (Fig. 8h). Analysis of Day 1 training trace data demonstrated an impairment in freezing percent in trace 3 for Tg mice treated with vehicle (p = 0.049) and no impairment present in Ntg mice or Tg mice treated with LM11A-31, suggesting a restoration of trace learning in the treated Tg mice. In day 2 context dependent testing, Tg mice exhibited a non-significant trend in decreased freezing behavior and treatment with LM11A-31 was associated with a non-significant trend toward normalization (Fig. 8i). On day 3 cue-based testing, Tg mice demonstrated a non-significant decrease in freezing behavior and treatment with LM11A-31 was associated with a non-significant trend for improvement.

## Discussion

The present study found that small molecule modulation of the p75^NTR^ receptor was associated with mitigation of a broad range of molecular mechanisms associated with tau pathology, including inhibition of excess activity of cdk5 and JNK tau kinases, excess calpain activity and deficient cofilin phosphorylation. There was a correspondingly wide effect on tau molecular status with reductions in tau isoforms detected through four phospho-epitopes, decreases in pathological tau-K280 acetylation, reduced tau misfolding and an apparent reduction in tau cleavage, with no effects on physiologic, native tau levels. Decreased accumulation of insoluble tau oligomers and filamentous forms was consistent with the reductions in these and potentially other PTMs. Diminished tau seeding activity was also found. These alterations in molecular pathology were associated with reductions in synaptic degeneration, normalization of dendritic trees and reversal of established deficits in dendritic spine density. In addition, two measures of microglial activation were found to be suppressed.

Finally, these changes in neurodegenerative and neuroinflammatory markers were associated with improvements in survival and several behavioral measures. In sum, we have found that small molecule-mediated modulation of p75^NTR^-coupled signaling networks produces significant therapeutic effects, can inhibit multiple aspects of the development of tau pathology and may even improve some morphological deficits. Moreover, these outcomes are observed in the setting of disease evoked by the expression and aggregation of mutant human tau, demonstrating the feasibility of strategies distinct from lowering endogenous tau levels or promoting tau clearance, to block formation/accumulation of such aggregates.

The proposed role for prion-like propagation of pathological tau species in AD and other tauopathies has spurred the development of therapeutic strategies that reduce levels of tau seeding activity [9, 17, 30]. Intracerebroventricular (ICV) infusion of tau antibodies into PS19 mice beginning at 6 months of age reduced AT8 staining, microglial activation and deficits in contextual fear behavior measured at 9 months of age [78] and lysates from treated mice demonstrated reduced tau seeding activity. Such antibodies are thought to bind to extracellular tau to block cellular uptake of tau aggregates, likely functioning through a reduction of extracellular cell-to-cell propagation with a secondary decrease in the formation of additional seeding activity in new locations. Challenges in this approach include the uncertain nature and potential diversity of the epitopes regulating seeding activity [11, 16] and limited access of extracellular antibodies to intracellular processes which may be fundamental drivers of propagation [66]. In another approach, ICV infusion of human tau-lowering antisense oligonucleotide (ASO) into PS19 mice beginning at 6 months of age led to reductions in transgene-expressed human tau mRNA and protein along with reduced tau aggregation, AT8 signal, ThioS staining and tau seeding activity [15]. Challenges for tau antisense approaches include adequate CNS distribution and potential side effects associated with chronically reduced physiological tau levels. The mechanisms of action described above are distinct from and may perhaps be complementary with a primary effect of preventing tau modifications that trigger seed formation as observed in the present study.

Loss of synaptic spines constitutes a fundamental pathological feature of AD [28] and other tauopathies including frontotemporal dementia [3, 19], though has been only recently studied in therapeutic studies of tauopathy mice. In a study from Chatterjee, et al. [7] treatment of 8-month old THY-Tau22 mice (G272V, P301S) with CSP-TTK21, a small molecule conjugated to a glucose-based carbon nanosphere that activates CBP/p300 histone acetyltransferase, was associated with increased hippocampal pyramidal neuron spine density. In the present study, LM11A-31 promoted an increase in spine density in Tg mice, in spite of a ~ 75% loss observed at 9 months of age, and provided a complete restoration of spine density. These findings suggest that fundamental tauopathy-related mechanisms underlying synaptic spine status are effectively engaged through modulation of p75^NTR^ signaling. The known interaction of p75^NTR^ with the RhoA GTPase/cofilin signaling module and the finding here that administration of LM11A-31 corrects the aberrant cofilin phosphorylation status present in PS19 mice, suggests that modulation of GTPase/cofilin signaling may be a component of these underlying mechanisms. Future studies will be important to further define such mechanisms and assess effects on spine dynamics, maturation and function.

In addition to effects on neurons, we found that LM11A-31-induced lowering of pathological tau modifications and species was associated with reduced signs of microglial activation. These findings are consistent with evidence that various tau aggregate forms are associated with activation of microglia in PS19 and other mouse models [42, 54, 71]. Another mechanism, not mutually exclusive, might involve modulation of microglial p75^NTR^ which is minimally expressed by normal microglia but can be upregulated in pathological settings [52].

The occurrence of multiple tau strains may pose further difficulties in therapeutic development [16, 65, 66]. Neurodegenerative properties have been associated with a broad range of oligomeric [24] and fibrillar [1, 40] tau forms. Therapeutic engagement of individual ‘upstream’ targets directly engaging tau such as kinases, acetylases or cleavage enzymes comprise important strategies but face the challenge that the formation of the gamut of toxic tau species, strains, and seeding activities, is likely a multifactorial process involving multiplex tau PTMs [23]. In addition, the efficacy of methods focused on blocking extracellular spread of *already*-*formed* tau seeding activity will likely be diminished by the persistent generation of that activity. The approach in the present study is distinct, in that specific tau isoforms or strains are not directly targeted, rather, a broad range of ‘upstream’ mechanisms contributing to manifold features of tau molecular pathology are engaged to reduce accumulation of seed-competent tau along with multiple measures of degeneration. This may increase the likelihood of affecting a robust spectrum of fundamental pathological processes in the human context and ultimately affect clinical outcomes in future trials.

## Supplementary information

**Additional file 1: Fig. S1.** LM11A-31 treatment reduces AT8 tau pathology in cortex. **a** AT8 immunostaining in 9-month old untreated and treated Ntg and Tg mice (AT8, red; Nissl staining, green). **b** % of cortical area with AT8-positive immunostaining in post-treatment mice. Statistical significance was determined using Kruskal–Wallis with post hoc Dunn’s testing; n = 6–11 mice per group, p-values are indicated.

**Additional file 2: Fig. S2.** Characterization of synaptosomal preparations. Crude/total protein hippocampal lysates and those same samples after synaptosomal enrichment from 3 Ntg and 3 Tg mice were probed for PSD95, LaminB1 and actin. LaminB1 was markedly reduced and relative amounts of PSD95 were increased, indicating enrichment in the synaptosomal component of each sample.

**Additional file 3: Fig. S3.** TauD1M9X and HT7 western blot shorter exposure times (reference Fig. 3e, j).

**Additional file 4: Fig. S4.** LM11A-31 treatment does not change PP2A or p62 protein levels. **a**, **b** Western blot analyses of hippocampal extracts were quantitated by determining the ratios of indicated protein to actin and normalized to Ntg control mice; n = 6–11 mice per group, with two independent western blots averaged per animal. **a** PP2A. **b** p62. Statistical significance was determined using one-way ANOVA. No significant changes were detected.

**Additional file 5: Fig. S5.** LM11A-31 treatment does not reduce GFAP expression. Western blots analysis of extracts of hippocampal extracts were quantitated by determining the ratios of GFAP to actin and normalized to Ntg control mice; n = 6–11 mice per group, with two independent western blots averaged per animal. Statistical significance was determined using ANOVA with post hoc Sidak’s multiple comparisons test, p-values are indicated.

## Data Availability

The datasets generated during and/or analyzed during the current study are available from the corresponding author on reasonable request.
