# Supplementary figures and images for: Small-molecule modulation of the p75 neurotrophin receptor inhibits a wide range of tau molecular pathologies and their sequelae in P301S tauopathy mice
Source: Acta Neuropathol Commun. 2020 Sep 5;8:156. doi: 10.1186/s40478-020-01034-0 (PMC7487850; doi:10.1186/s40478-020-01034-0)

Supplementary Figure 1

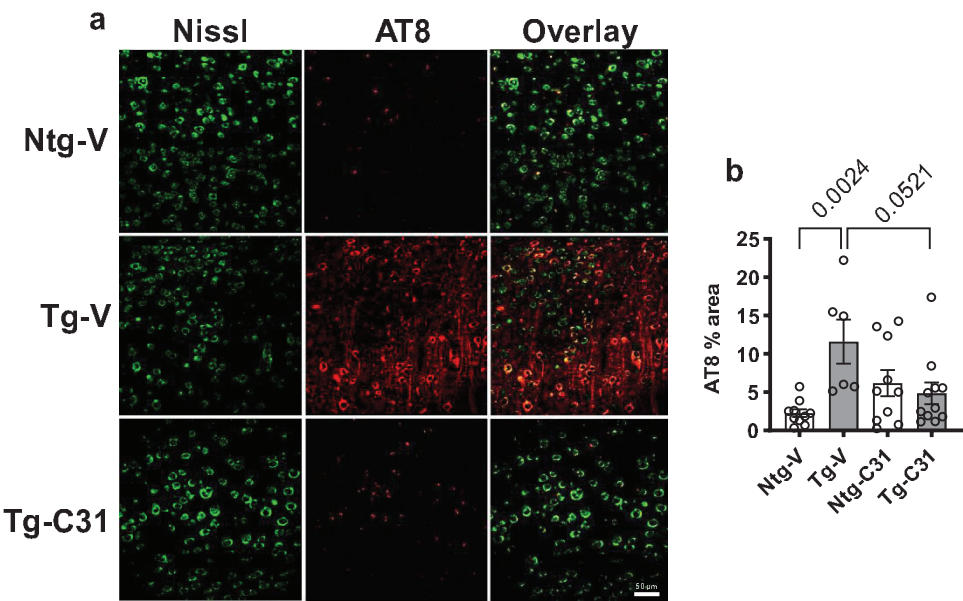

Supplement: Supplementary file 1 — Additional file 1: Fig. S1. LM11A-31 treatment reduces AT8 tau pathology in cortex. a AT8 immunostaining in 9-month old untreated and treated Ntg and Tg mice (AT8, red; Nissl staining, green). b % of cortical area with AT8-positive immunostaining in post-treatment mice. Statistical significance was determined using Kruskal–Wallis with post hoc Dunn’s testing; n = 6–11 mice per group, p-values are indicated. [file 40478_2020_1034_MOESM1_ESM.pdf]

## Supplementary Figure 2

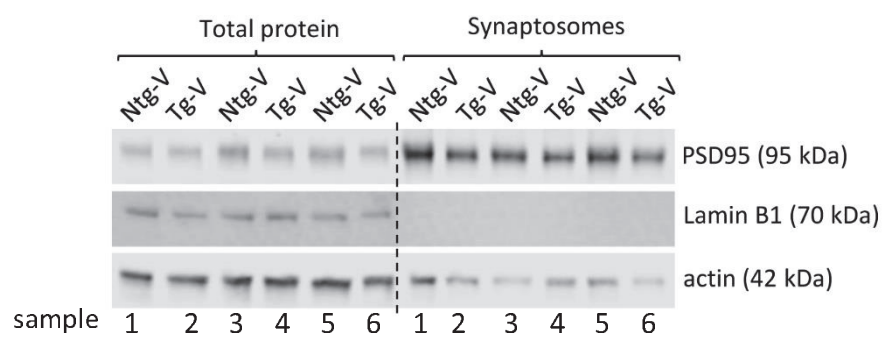

Supplement: Supplementary file 2 — Additional file 2: Fig. S2. Characterization of synaptosomal preparations. Crude/total protein hippocampal lysates and those same samples after synaptosomal enrichment from 3 Ntg and 3 Tg mice were probed for PSD95, LaminB1 and actin. LaminB1 was markedly reduced and relative amounts of PSD95 were increased, indicating enrichment in the synaptosomal component of each sample. [file 40478_2020_1034_MOESM2_ESM.pdf]

Supplementary Figure 3

TauD1M9X and HT7 shorter exposure times (reference Figure 3e, j)

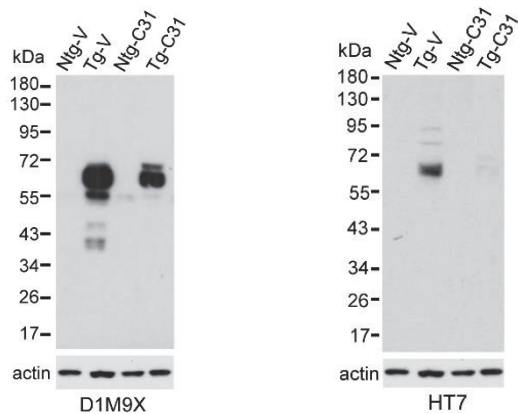

Supplement: Supplementary file 3 — Additional file 3: Fig. S3. TauD1M9X and HT7 western blot shorter exposure times (reference Fig. 3e, j). [file 40478_2020_1034_MOESM3_ESM.pdf]

Supplementary Figure 4

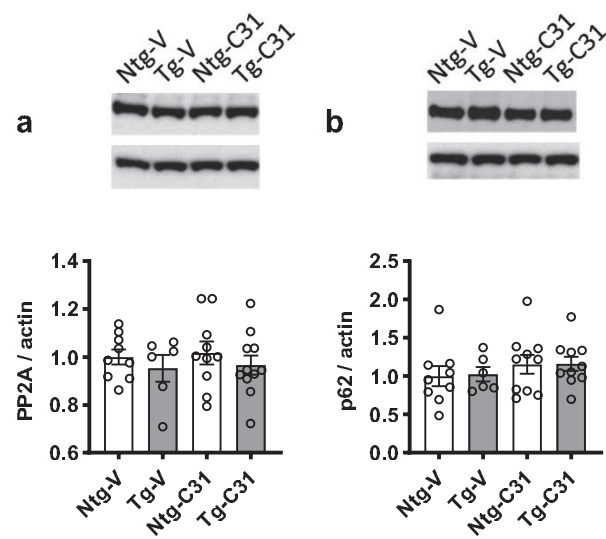

Supplement: Supplementary file 4 — Additional file 4: Fig. S4. LM11A-31 treatment does not change PP2A or p62 protein levels. a, b Western blot analyses of hippocampal extracts were quantitated by determining the ratios of indicated protein to actin and normalized to Ntg control mice; n = 6–11 mice per group, with two independent western blots averaged per animal. a PP2A. b p62. Statistical significance was determined using one-way ANOVA. No significant changes were detected. [file 40478_2020_1034_MOESM4_ESM.pdf]

Supplementary Figure 5

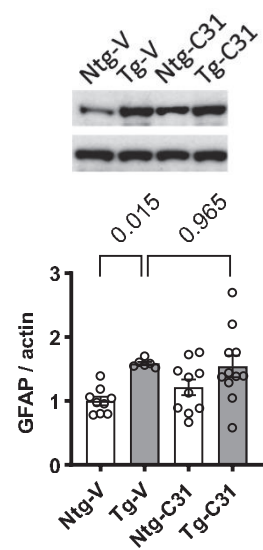

Supplement: Supplementary file 5 — Additional file 5: Fig. S5. LM11A-31 treatment does not reduce GFAP expression. Western blots analysis of extracts of hippocampal extracts were quantitated by determining the ratios of GFAP to actin and normalized to Ntg control mice; n = 6–11 mice per group, with two independent western blots averaged per animal. Statistical significance was determined using ANOVA with post hoc Sidak’s multiple comparisons test, p-values are indicated. [file 40478_2020_1034_MOESM5_ESM.pdf]
